# N6-methyladenosine modulates long non-coding RNA in the developing mouse heart

**DOI:** 10.1038/s41420-022-01118-x

**Published:** 2022-07-20

**Authors:** Siman Shen, Keyu Liu, Simeng Li, Sanketh Rampes, Yuhui Yang, Yifeng Huang, Jing Tang, Zhengyuan Xia, Daqing Ma, Liangqing Zhang

**Affiliations:** 1 Department of Anesthesiology, Affiliated Hospital of Guangdong Medical University, Zhanjiang, China; 2 Key Laboratory of Organ Functional Injury and Protection and Department of Translational Medicine of ZhanJiang, ZhanJiang, China; 3 Division of Anaesthetics, Pain Medicine and Intensive Care, Department of Surgery and Cancer, Faculty of Medicine, Imperial College London, Chelsea and Westminster Hospital, London, UK

**Keywords:** Developmental biology, Cardiovascular diseases

## Abstract

Long non-coding RNAs (lncRNAs) were reported to potentially play a regulatory role in the process of myocardial regeneration in the neonatal mouse. N6-methyladenosine (m^6^A) modification may play a key role in myocardial regeneration in mice and regulates a variety of biological processes through affecting the stability of lncRNAs. However, the map of m^6^A modification of lncRNAs in mouse cardiac development still remains unknown. We aimed to investigate the differences in the m^6^A status of lncRNAs during mouse cardiac development and reveal a potential role of m^6^A modification modulating lncRNAs in cardiac development and myocardial regeneration during cardiac development in mice. Methylated RNA immunoprecipitation sequencing (MeRIP-seq) and RNA sequencing (RNA-seq) of the heart tissue in C57BL/6 J mice at postnatal day 1 (P1), P7 and P28 were performed to produce stagewise cardiac lncRNA m^6^A-methylomes in a parallel timeframe with the established loss of an intrinsic cardiac regeneration capacity and early postnatal development. There were significant differences in the distribution and abundance of m^6^A modifications in lncRNAs in the P7 vs P1 mice. In addition, the functional role of m^6^A in regulating lncRNA levels was established for selected transcripts with METTL3 silencing in neonatal cardiomyocytes in vitro. Based on our MeRIP-qPCR experiment data, both lncGm15328 and lncRNA *Zfp597*, that were not previously associated with cardiac regeneration, were found to be the most differently methylated at P1-P7. These two lncRNAs sponged several miRNAs which further regulated multiple mRNAs, including some of which have previously been linked with cardiac regeneration ability. Gene Ontology and Kyoto Encyclopedia of Genes and Genomes analysis revealed that differential m^6^A modifications were more enriched in functions and cellular signalling pathways related to cardiomyocyte proliferation. Our data suggested that the m^6^A modification on lncRNAs may play an important role in the regeneration of myocardium and cardiac development.

## Abstract (web summary)

The graphical abstract of the potential mechanism of m^6^A modulates long non-coding RNA in the developing mouse heart.

## Graphical abstract

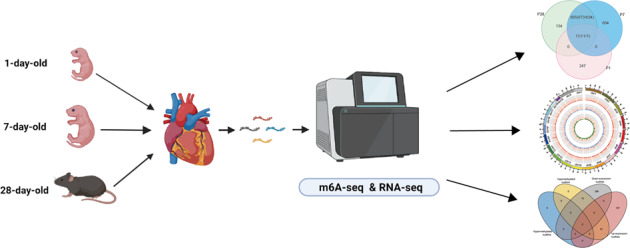

## Introduction

The heart is a terminally differentiated organ and it has been accepted that the heart lacks the ability to regenerate [1, 2]. Notably, excellent heart functions and long-term outcomes in human newborns and neonates/toddles were noted following treatment or surgery from massive cardiac infarction [3] or congenital heart disease [4], respectively, indicating that the young heart may have some regenerating capability. However, self-renewal of cardiomyocytes in humans, and that the renewal rate of cardiomyocytes decreases with age [5, 6]. The myocardium of the postnatal day 1 (P1) mouse can be completely regenerated after surgical resection of the apex of heart, this regenerative ability is lost at P7 [7, 8]. Although the underlying mechanisms of the lost ability remain unknown, these including cardiac polyploidy, multi-levelled early innate immune system, “cancer risk” suppression and cardiac thyroxin signaling activation may likely be a barrier for cardiomyocyte proliferation [9–13]. Thus, studying the changes of myocardial regeneration ability during the neonatal-to-adult mouse heart transition may help to elucidate the mechanism of myocardium regeneration.

N6-methyladenosine (m^6^A) is the most prevalent internal modification in eukaryotic cells [14]. m^6^A modification reflects a dynamic and reversible process, which involves methyltransferases (METTL3, METTL14 and WTAP), demethylases (FTO, ALKBH5 and ALKBH3) and RNA-binding proteins (YTHDF1-3, YTHDC1-2 and IGF2BPs) processing [15]. A previous study showed that METTL3 was downregulated in the postnatal day (P7) relative to P0 heart in rat newborns, and enhancing METTL3 expression level of P0 cardiomyocytes resulted in increased proliferation in vitro[16]; however, in opposition to this, a recent study showed identified global METTL3 knockout enhanced cardiac regeneration and repair after myocardial injury [17]. It was also reported that m^6^A methylation was significantly increased and the expression level of ALKBH5 was decreased after birth in the mouse heart and its overexpression promoted myocardial regeneration and repair after the myocardial infarction (MI) [18]. m^6^A methylation was reported to be the key regulator of cardiac development and myocardial regeneration [18, 19], but the underlying mechanisms remain elusive.

LncRNAs are a class of special RNA transcripts with more than 200 nucleotides and are widely known to participate in various biological and pathological processes [20, 21]. Myocardial regeneration is regulated by lncRNAs [22–24]. The lncRNA CAREL was upregulated in the P7 mouse heart in parallel with loss of cardiac regenerative capacity. Thus, lncRNA CAREL overexpression in cardiomyocytes inhibited its proliferation, whilst silencing it in the heart promoted myocardial regeneration and improved cardiac function in the infarcted area [25]. lncRNA ECRAR expression was the highest in the P1 rat heart and then was progressively decreased in rat hearts after birth; its overexpression not only promoted myocardial regeneration but also improved post-infarction cardiac function [26]. Taken together, current evidence strongly suggests that cardiomyocyte proliferation can be triggered by a variety of endogenous factors.

Here, we performed a m^6^A-specific analysis and related bioinformatics analysis of m^6^A on lncRNAs from the heart tissue of C57BL/6 J mice at P1, P7, and P28. These approaches produced multiple datasets describing cardiac lncRNA m^6^A methylomes from different developmental stages early after birth which appear in a parallel timeframe with the established loss of intrinsic cardiac regeneration capacity. Thus, our work reported here may open avenues for identifying novel regeneration-related lncRNAs and molecular pathways regulated by m^6^A.

## Results

### Motif analysis

**Fig. 1:**
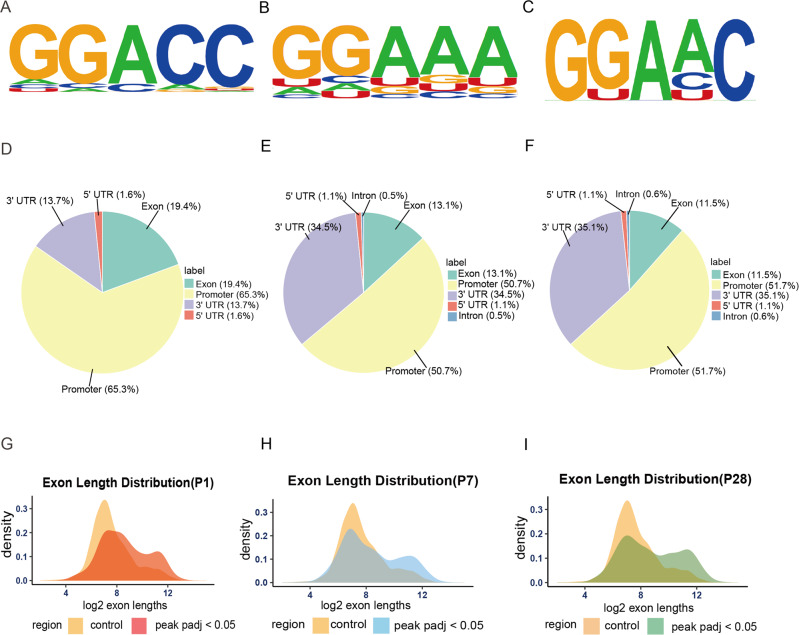
Distribution of m^6^A peaks for host genes of lncRNAs. **A**–**C** top m^6^A motifs enriched from all identified m^6^A peaks in postnatal day 1 (P1), P7, P28 heart tissue; *P* < 0.05 (*n* = 3) (hypergeometric test). **D**–**F** Pie chart showing the distribution profiles of m^6^A peaks for host genes of lncRNA in P1, P7, P28 heart tissue. **G**–**I** The distribution of m^6^A peaks on exon length; Adjusted p values with False Discovery Rate; *p* < 0.05 (*n* = 3) (hypergeometric test). (control: lncRNAs without m^6^A peaks; peak: lncRNAs with m^6^A peaks).

HOMER software was applied to map the myocardial lncRNA m^6^A methylomes (P1, P7 and P28). RRACH (R = G or A; H = A, C or U) is the most conserved sequence motif in P1 (Fig. 1A, *P* < 0.05), which is in line with the previous studies [27, 28]. Meanwhile, P7 conserved motif is RRA (A/N)(A/N), and P28 conserved motif is RRAHC (Fig. 1B, C, *P* < 0.05).

### Distribution of m^6^A peaks for host genes of lncRNAs

We analyzed the data and plotted pie charts to identify the distribution profiles of m^6^A peaks for host genes of lncRNAs (Fig. 1D–F). Most m^6^A peaks were enriched in the promoter region, followed by 3′ UTR region. During the development of the mouse heart, m^6^A peaks showed great changes in the promoter region between P1 (65.3%) and P7 (50.7%), but there was a marginal difference between P7 (50.7%) and P28 (51.7%) groups. Additionally, the exon length of lncRNA with m^6^A peaks had no significant difference among the three groups (Fig. 1G–I, Supplemental Fig. S1). These data suggested that from day 1 to 7 after birth, the distribution of m^6^A peaks underwent a major reorganization in a short period of 7 days, while the lncRNA m^6^A methylome remained relatively stable in terms of m^6^A peak distribution across lncRNAs after P7.

### Abundance features of m^6^A peaks on lncRNAs and Chromosomes

**Fig. 2:**
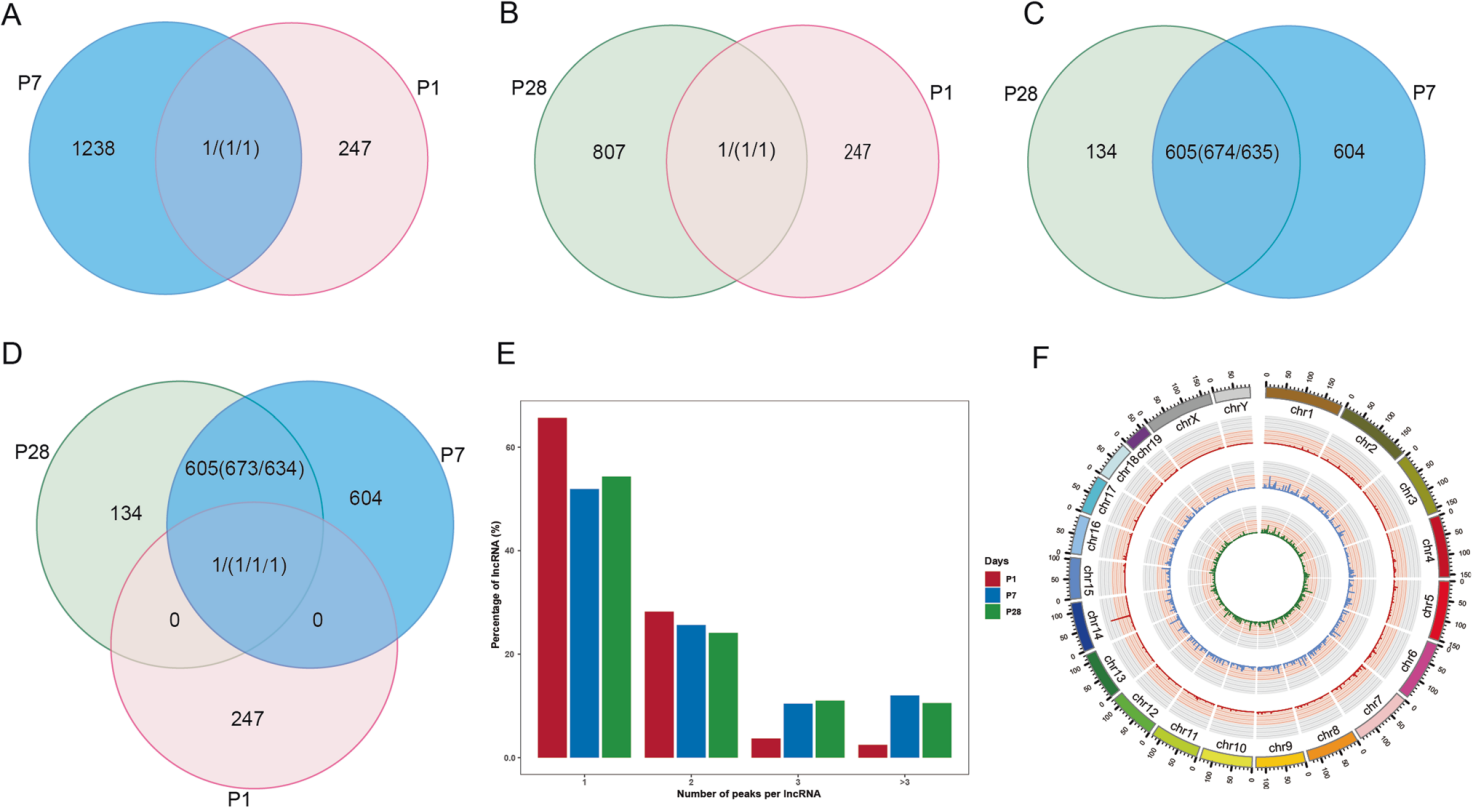
Abundance features of m^6^A peaks on lncRNAs and chromosomes. **A** Venn diagram showing the overlap of m^6^A peaks within lncRNAs in postnatal day 7 (P7) and P1 heart tissue. **B** Venn diagram showing the overlap of m^6^A peaks within lncRNAs in P28 and P1 heart tissue. **C** Venn diagram showing the overlap of m^6^A peaks within lncRNAs in P28 and P7 heart tissue. **D** Venn diagram showing the overlap of m^6^A peaks within lncRNAs in P1, P7 and P28 heart tissue. **E** Percentage of lncRNAs harboring different m^6^A peaks in these three groups, it seems that most of the methylated lncRNAs harbring only one m^6^A peak. **F** Circos plot showing the distribution of the m^6^A sites within lncRNAs on each chromosome in P1, P7 and P28 heart tissue (red: P1; blue: P7; green: P28).

To gain insight into transcriptome-wide m^6^A methylation, m^6^A-sequencing of lncRNAs in C57BL/6 J mouse heart tissue of P1, P7, and P28 was performed. lncRNAs m^6^A peaks were to be 248 at P1, increased to 1,239 at P7 and decreased to 808 at P28. The differences and overlaps of m^6^A peaks among these three groups are displayed in a Venn diagram, and some redundancy data were merged in a statistical mapping (group P1 were compared with group P7, and one peak in P1 overlapped with two or more peaks in P7, namely redundancy). Among them, only 1 m^6^A peak (host gene name: *Aopep*, Aminopeptidase O) was common between the P1 and P7 mice, and this single conserved m^6^A lncRNA peak across all study groups (Fig. 2A, B, D). However, there are 605 overlapping m^6^A peaks between P7 and P28 (Fig. 2C). The majority of lncRNAs contained only one m^6^A peak (more than 50%), and this ratio is highest at P1 (P1:66.5%, P7:52.1%, P28:56.4%) (Fig. 2E). Towards adolescence, the m^6^A in lncRNAs within mouse myocardium was increased in stoichiometry, but the datasets showed that multiply methylated lncRNAs was not associated with different expression levels. Circos software was used to analyze the distribution of lncRNA methylation sites on the chromosomes (Fig. 2F). It can be seen that there was a disappearance of the highly prominent P1 lncRNA m^6^A peak at chromosome 14 around *Myh6*-*Myh7* host genes by the P7 and P28.

### Correlation analysis between m^6^A modifications and lncRNAs expression

**Fig. 3:**
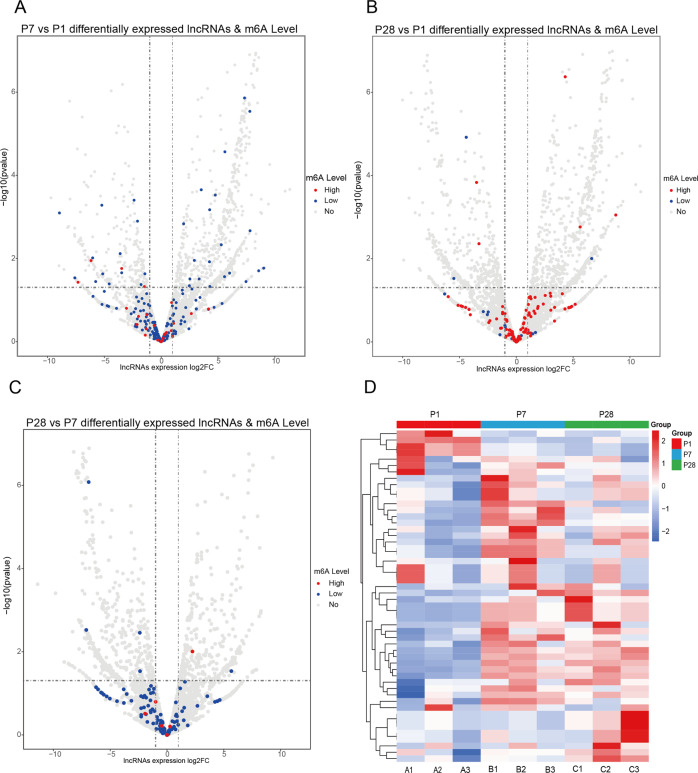
Correlation analysis between m^6^A modifications and lncRNAs expression. **A**–**C**. The relationship between lncRNAs expression level and methylation level; *P* < 0.05 (*n* = 3) (Student t-test), Red dot (High): m^6^A level increased (FC > 1.5, *P* < 0.05), Blue dot (Low): m^6^A level decreased (FC > 1.5, *P* < 0.05), Gray dot (No): m^6^A level variation: |FC | < 1.5. **D** Cluster analysis of m^6^A in postnatal day 1 (P1), P7 and P28 heart tissue, red color for hypermethylated and blue for hypomethylated. The gradient color legend unit indicates the fold change number of m^6^A, and use the pheatmap function of the pheatmap package in R to normalize the fold change number.

To uncover whether the differentially expressed lncRNAs were associated with m^6^A methylation changes, we performed the cross-analysis of the RNA-Seq and m^6^A-Seq data. The upregulated lncRNAs solely were either hypomethylated or not methylated at all, while the downregulated lncRNAs were either hypermethylated, hypomethylated or not methylated when comparing P7 to P1 (Fig. 3A). These indicated that the methylation-status associated poorly with downregulation of lncRNA expression whilst the lncRNA hypomethylation was associated more consistently with the respective transcript overexpression. However, in comparisons with P28 vs P1 and P28 vs P7 together, there was a non-existent correlation with the lncRNA expression (Fig. 3B, C). To further assess the m^6^A methylation levels difference of lncRNAs in these three developmental stages of mouse heart tissue, the clustering of methylation differences were clearly be distinguished in the P1, P7 and P28 mouse heart (Fig. 3D). The differentially methylated and expressed lncRNA transcripts were more numerous in P7 vs P1, indicating that m^6^A modification might be associated with and even regulate lncRNA expression especially in P1 to P7.

Among the 40 lncRNAs with the largest methylation modified fold changes (Supplemental Table S2-5), some of these lncRNAs had big differences in m^6^A methylation such as lncRNA *Myh7*. Interestingly, *Myh7* gene was reported to be a classic biomarker for cardiac development and a potential target for attenuating cardiomyocyte hypertrophy [29, 30]. However, lncRNA *Myh7* was not changed in our study which may indicate that the change of lncRNA *Myh7* m^6^A methylation during P1-P28 was unlikely converted to the altered itself expression.

### m^6^A RNA methylation-related lncRNAs (m6A-lncRNAs) may have potential effect on heart development

**Fig. 4:**
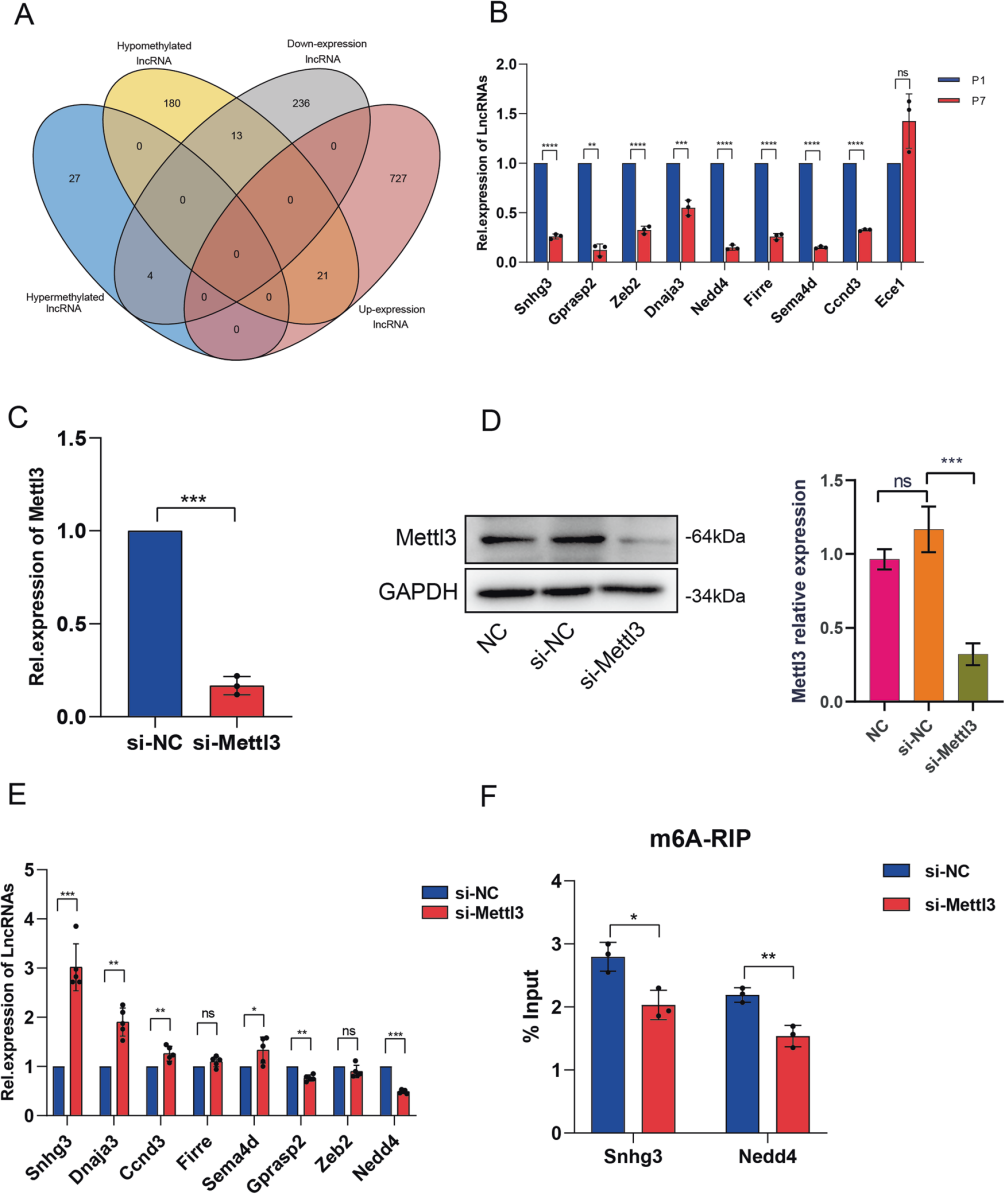
m^6^A RNA methylation-related lncRNAs (m^6^A-lncRNAs) may have potential effect on heart development. **A** Veen diagram of lncRNAs of different expression and methylation including up-regulated and down-regulated between postnatal day 1 (P1) and P7. **B** RT-qPCR assay was applied to determine the expression of 9 genes related to cardiac disease. **C** RT-qPCR analysis of METTL3 in cultured cardiomyocytes transfected with siRNA and negative control siRNA (si-NC). **D** Western blot analysis of METTL3 of cardiomyocytes transfected with siRNA and siRNA-ctrl, quantitated by Image J. **E** The expression of lncRNAs by RT-qPCR in siRNA-transfected cardiomyocytes, Data expressed as mean ± SEM (*n* = 5); *P < 0.05 (Student t-test). **F** MeRIP-qPCR results of lncRNA *Snhg3* and lncRNA *Nedd4* transfected with si-Mettl3 of cardiomyocytes, showing the m^6^A levels of lncRNA *Snhg3* and lncRNA *Nedd4* in the way of bounding with anti-m^6^A antibody, Data expressed as mean ± SEM (*n* = 3); *P < 0.05 (Student t-test). %input means the percentage of genes that has been methylated. %input=2^(-ΔCt normalized RIP)^. n.s.: *P* > 0.05; *P < 0.05; **P < 0.01; ***P < 0.001.

By cross-analysis of the m^6^A-Seq and RNA-seq data, a total of 38 lncRNAs was found be with expression changes closely related to m^6^A modifications in the P7 vs P1 group (Fig. 4A and Supplemental Table S6).

To explore the impact of m^6^A modifications on lncRNAs expression in mouse heart development, 9 of these 38 lncRNAs **(**Supplemental Table S1) were found either to be related to myocardial development or regeneration, or otherwise, may be the new molecules that potentially regulates myocardial regeneration. Eight of 9 lncRNAs expression in P1 and P7 mouse heart tissue were consistent with qPRCR sequence (Fig. 4B). It is known that METTL3 is the catalytic subunit responsible for the m^6^A writing by the MTC (methyltransferase complex) [15]. Additionally, METTL3 mediated m^6^A methylation within myocardium plays an indispensable role in cardiac homeostasis especially in aging [19]. We then used siRNA targeting METTL3 to reduce the endogenous expression of METTL3 in neonatal mouse cardiomyocytes (NMCMs) (Fig. 4C, D). As shown in Fig. 4E, knockdown of METTL3 in NMCMs, among the lncRNAs with changes in expression, lncRNA *Snhg3* was the most significant increase (Fold Change = 3.02) and lncRNA *Nedd4* was the most significant decrease. Meanwhile, m^6^A modification of ‘lncRNA *Snhg3*’ and ‘lncRNA *Nedd4*’ were decreased upon METTL3 knockdown as shown by MeRIP-qPCR assay (Fig. 4F), suggesting that these two lncRNAs may be the target of METTL3. Moreover, the Nedd4 has earlier been reported to be a potential key factor for myocardial regeneration [31].

**Fig. 5:**
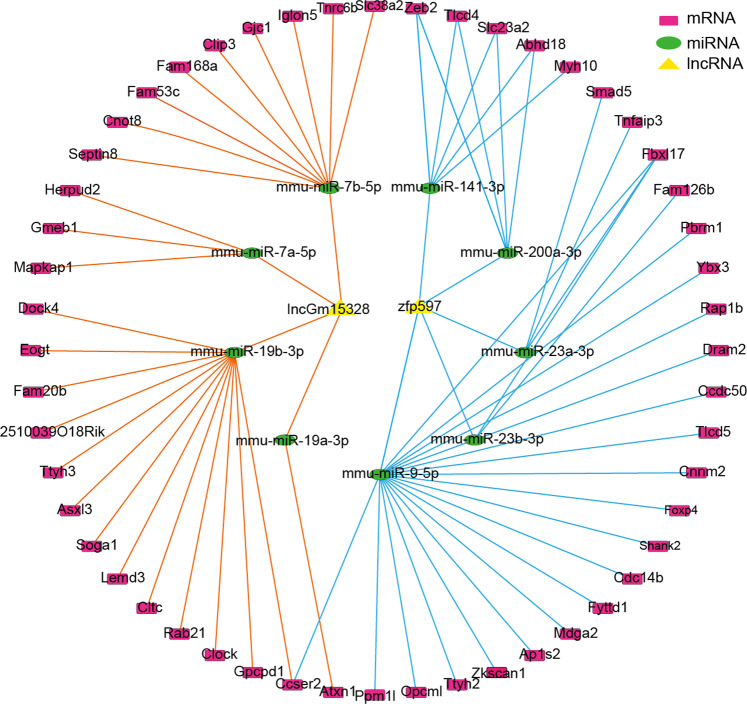
lncRNA-miRNA-mRNA network of *lncGm15328* and lncRNA *Zfp597*. The red rectangles = mRNAs; the green ellipses = miRNAs; the yellow triangles =lncRNAs(lncGm15328 and lncRNA *Zfp597*). The line represents a co-expression relationship between the lncRNA and the miRNA or the miRNA and the mRNA.

lncRNAs can act as microRNA (miRNA) sponges in regulating protein-coding gene expression [32]. The lncRNA-miRNA-mRNA regulatory network plays critical role in cardiac regeneration [33]. Among the 38 lncRNAs, two lncRNAs (*LncGm15328*: the largest increase of m^6^A modification; lncRNA *Zfp597*: the largest decrease of m^6^A modification) with the biggest difference m^6^A modification were selected to establish a ceRNA network (Fig. 5). The lncRNA-miRNA-mRNA network *LncGm15328* and lncRNA *Zfp597* was related to the target of miR-19a/19b and miR-9, respectively. Existing literatures reported that miR-19a/19b was directly involved in regulating myocardial regeneration, and miR-9 was shown to be correlated with cardiogenin-treated regenerating heart [34, 35].

### Gene ontology and Kyoto Encyclopedia of Genes and Genomes analyses of lncRNAs harboring differentially methylated N6-methyladenosine sites

To investigate the function of differentially methylated lncRNAs in P1, P7, and P28 heart tissue, we performed Gene Ontology (GO) enrichment analysis, which contains biological processes (BP), cellular components (CC), and molecular functions (MF). Kyoto Encyclopedia of Genes and Genomes (KEGG) analysis was used to identify pathways in which differentially methylated lncRNAs may be involved. There was little difference in m^6^A modification of lncRNAs between P7 and P28 group, so we focused on P7 vs P1 and P28 vs P1 for analysis.

**Fig. 6:**
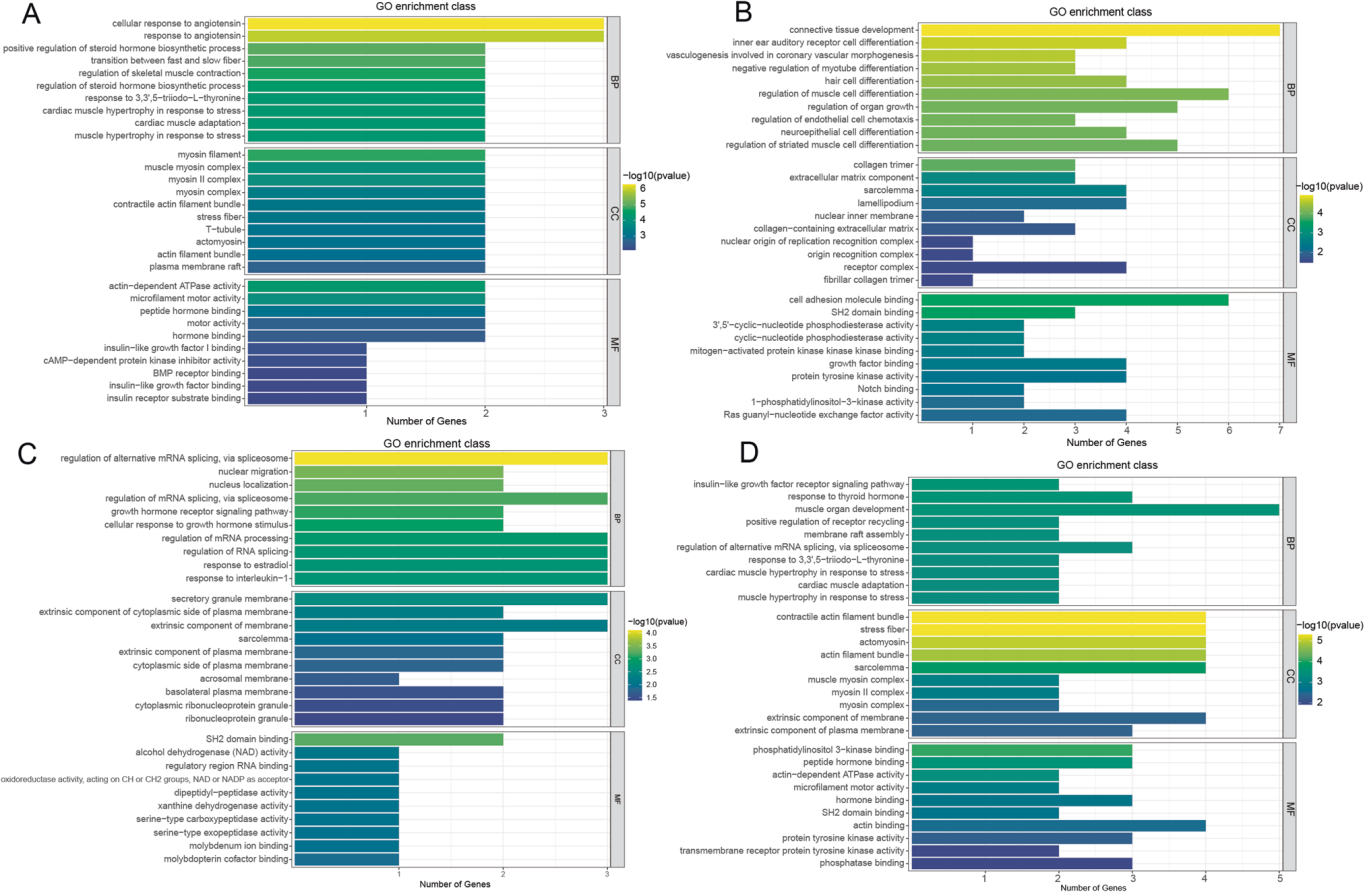
Gene Ontology (GO) analyses of lncRNAs harboring differentially methylated N6-methyladenosine sites. **A** Top ten GO terms of biological processes(BP), cellular components (CC) and molecular functions (MF) were significantly enriched for the hypermethylated lncRNAs in postnatal day 7 (P7) vs P1. **B** Top ten GO terms of BP, CC and MF were significantly enriched for the hypomethylated lncRNAs in P7 vs P1. **C** Top ten GO terms of BP, CC and MF were significantly enriched for the hypermethylated lncRNAs in P28 vs P1. **D** Top ten GO terms of BP, CC and MF were significantly enriched for the hypomethylated lncRNAs in P28 vs P1; *P* < 0.05 (*n* = 3) (Hypergeometric test).

**Fig. 7:**
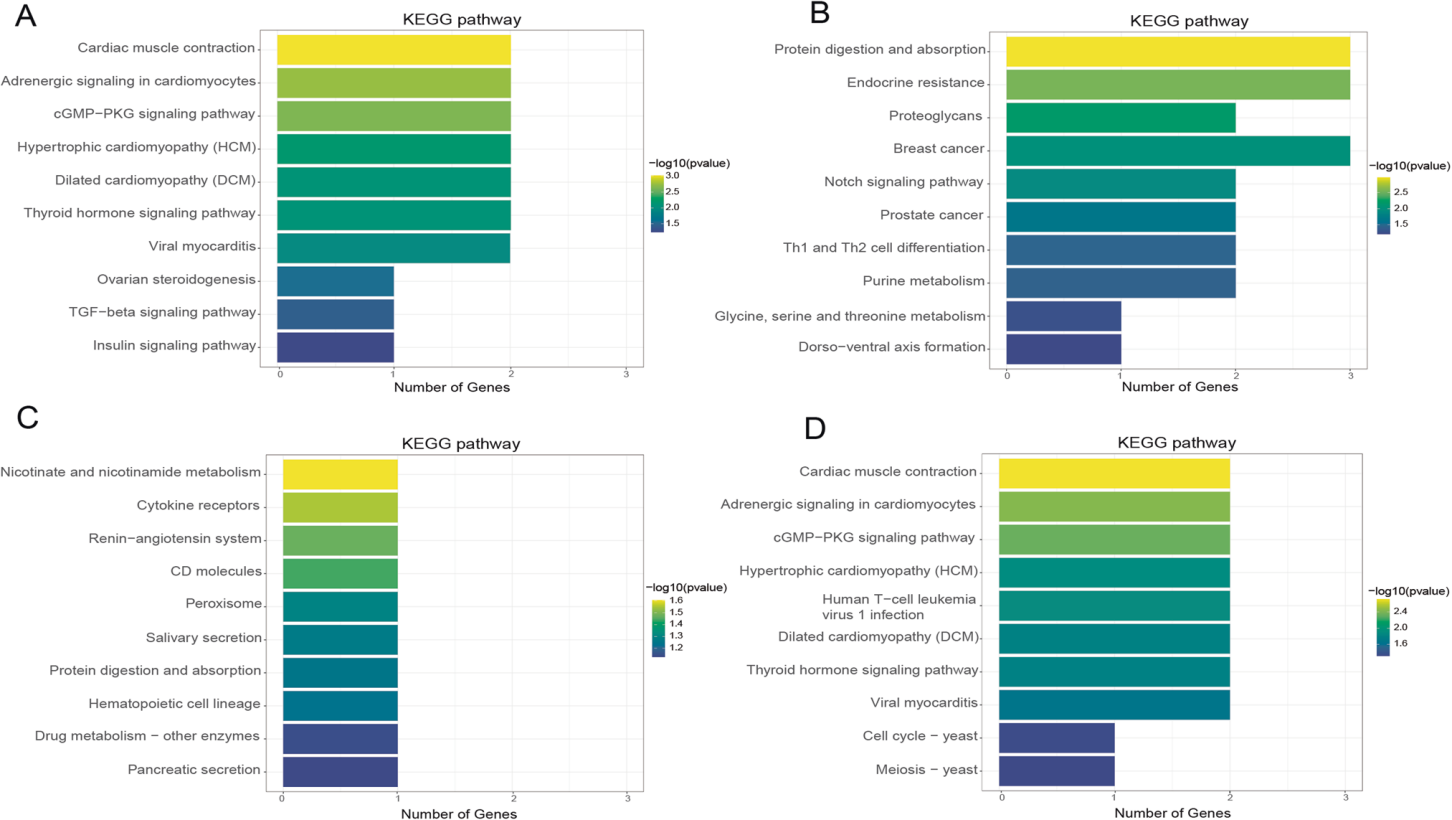
Kyoto Encyclopedia of Genes and Genomes (KEGG) analyses of lncRNAs harboring differentially methylated N6-methyladenosine sites. **A** Bar plot showing the top ten significant enrichment pathway for the hypermethylated lncRNAs in postnatal day 7 (P7) vs P1. **B** Bar plot showing the top ten significant enrichment pathway for the hypomethylated lncRNAs in P7 vs P1. **C** Bar plot showing the top ten significant enrichment pathway for the hypermethylated lncRNAs in P28 vs P1. **D** Bar plot showing the top ten significant enrichment pathway for the hypomethylated lncRNAs in P28 vs P1.

P7 vs P1: GO enrichment analysis showed that lncRNAs with up-methylated m^6^A sites were mainly enriched in the cellular response to angiotensin, myosin filament and actin-dependent ATPase activity, whereas lncRNAs with down-methylated sites were mostly enriched in connective tissue development, regulation of organ growth and growth factor binding (Fig. 6A, B). KEGG analysis showed that lncRNAs with up-methylated m^6^A sites were highly related to ‘Cardiac muscle contraction’ and ‘Adrenergic signaling in cardiomyocytes’. However, lncRNAs with down-methylated sites were significantly enriched in ‘Protein digestion and absorption’ (Fig. 7A, B).

P28 vs P1: GO enrichment data showed that lncRNAs with up-methylated m6A sites were especially enriched in regulation of alternative mRNA splicing via spliceosome, secretory granule membrane and SH2 domain binding. Impressively, lncRNAs with down-methylated sites were especially enriched in insulin-like growth factor receptor signaling pathway and response to thyroid hormone (Fig. 6C, D). It has been demonstrated that the insulin-like growth factor pathway is intricately related to hypertrophy [36] and thyroid hormone was responsible for murine MYH7 (beta-MHC, myosin heavy chain)-to-MYH6 (alpha-MHC) cardiac myosin isoform switch in early postpartum development [37].

Moreover, data from KEGG analysis showed that ‘Nicotinate and nicotinamide metabolism’ and ‘Renin-angiotensin system’ were significantly enriched in lncRNAs with up-methylated m^6^A sites. The ‘Nicotinate and nicotinamide’ are intricately related, among others, to glycolysis. Previous studies have reported that nicotinamide can stimulate glycolysis in cardiomyocytes [38], and glycolysis was heavily associated with cardiac regenerative ability within “hypoxic cardiomyocyte niches” [39]. Notably, postpartum relative hyperoxia inhibited glycolysis leading to halt regenerative ability [40], while hypoxia-induced anaerobic metabolism elevated glycolysis to promote regenerative milieu within the myocardium [41–43]. Angiotensin was known to promote interstitial cardiac fibrosis and hypertrophy [44]. In contrast, lncRNAs with down-methylated sites were involved in ‘Cardiac muscle contraction’ and ‘Cell cycle-yeast’ (Fig. 7C, D).

## Discussion

It has been established that the mouse myocardium poses an inherent capacity for major regeneration. However, this capacity is operative only during the first week after birth [7]. After which, its regenerative capability was lost and that may likely be due to the underlying mechanisms of ex utero relative hyperxia-induced DNA damage, cardiac polyploidy, multi-levelled early innate immune system, “cancer risk” suppression mechanism and cardiac thyroxin signaling activation [9–13, 40]. Interestingly, m^6^A modification, as the most common modification in mRNA and lncRNA, is involved in a variety of biological processes [14, 15]. However, m^6^A modification within lncRNA in the neonatal mouse heart still remains unexplored territory. In this study, we performed m^6^A-seq and RNA-seq to sequence the genome-wide profiling of methylation-modified lncRNAs in the heart of P1, P7 and P28 mice, investigated differences between these three developmental stages and found a potential role for m^6^A methylation of lncRNAs in cardiac development.

During myocardial injury, such as myocardial infarction (MI), cardiomyocytes in extremis may be highly susceptible to arrhythmias due to metabolic disturbances, and necrosis may lead to acute cardiac dysfunction. Then fibrosis of the damaged area occurs in the following days to weeks, which, depending on the extent of the resulting scar, often leads to heart failure as a late-stage complication [45]. For example, there are 2 to 4 billion (3.2 ± 0.75 billion) cardiomyocytes in the human left ventricle [6], and MI can destroy 25% of these cardiomyocytes depending on the occlusion site and revascularization time [46]. Therefore, identifying methods to promote the proliferation of cardiomyocytes in the area where cardiomyocytes are lost is an urgent medical need. Activating proliferation of cardiomyocytes may be an attractive approach to repair myocardial injury caused by MI or other heart diseases [47]. Although regenerative approaches are receiving significant attention, the mechanism underpinning the regulation of myocardial regeneration is complex and still remains incompletely understood.

m^6^A modification changes appear dynamically at different stages of organ development, especially in mammalian hearts, suggesting that m^6^A methylation plays an important role in heart growth [48, 49]. Additionally, accumulating evidence showed that the m^6^A level is closely related to the development of heart disease; for example, METTL3 overexpression can induce eccentric remodeling and cardiac dysfunction alone without an additional stressor [19]. m^6^A is also involved in the process of myocardial regeneration [16–18]. It has previously been documented that compared with normal heart tissue, the level of m^6^A modification is higher and FTO expression level is lower in myocardium tissue of mice with ischemic damage and heart failure [50]. In a MI mouse model, FTO overexpression can reduce myocardial fibrosis, enhance angiogenesis, and improve cardiac function [50]. Another interesting new finding was that m^6^A modification enhanced the recruitment of miR-133a-RISC (RNA-induced silencing complexes)-AGO2 (Argonaute 2-Insulin-like growth factor 2) to its targets in heart development and in response to cardiac hypertrophy and proliferation [51]. The above evidence presented thus far supports the idea that m^6^A may become a new target for the treatment of cardiovascular diseases in the future. There is already strong evidence of the importance of lncRNAs in the regulation of cardiomyocyte proliferation. Indeed, lnc CPR and Sirt1 AS lncRNA has previously been shown to regulate cardiomyocyte proliferation in vitro and vivo [52, 53]. The effects of two lncRNAs lnc CAREL and lnc DACH1 in cardiomyocytes proliferation were also investigated in induced pluripotent stem cells (iPSC)-derived cardiomyocytes [25, 54]. Nevertheless, whether m^6^A modification can regulate myocardial regeneration through controlling the expression of myocardial regeneration related lncRNAs still remains unknown. Therefore, exploring the interaction between m^6^A and lncRNAs in cardiac development will improve our understanding the mechanism of myocardial regeneration.

We identified methylation peaks on lncRNAs and found significant differences in the distribution and abundance of methylation peaks between P1 and P7, but such difference was not detectable at the later stage after P7 at P28. Indeed, here we found that the most prominent reorganization in the m^6^A peak distribution within lncRNAs occurred at across the 3′UTR and promoter regions. Namely, the relative fraction of m^6^A peaks from the promoter region at P1 was reduced and the fraction of m^6^A peaks at 3′UTR was increased at P7. Considering that the m^6^A in 3′UTR has been shown to regulate the stability of the respective RNA [55], the difference in the distribution of m^6^A modification in the 3′UTR region may affect the stability of lncRNAs during heart development to control myocardial regeneration ability. Recent studies have reported that lncRNAs harboring open reading frame (ORF) sequences can encode proteins/peptides [56]. For example, LINC00998 was originally described as a non-coding transcript, but a 180 nucleotide (nt) smORF (small ORF) was found in exon 3, which encoded a small integral membrane protein 30 (SMIM30) [57]. However, we discovered that the length of the exons contained in the m^6^A-modified lncRNA has no significant difference during heart development. Therefore, we speculated that the m^6^A modification may act principally by regulating the stability of lncRNAs, but has little effect on the protein-encoding process of lncRNAs. The exact m^6^A reader involved in the stabilization and degradation of lncRNA and the functional role of these several unveiled m^6^A modified lncRNA are unknown and all these need to be explored further in vivo cardiac regeneration models in the future. In addition to the distribution of m^6^A methylation, there are also great difference in the m^6^A abundance of the P1, P7 and P28 heart. Indeed, we noticed a near-total reorganization of m^6^A peaks within lncRNAs from P1 to P7 myocardium with only one conserved peak within lncRNA *Aopep*. In addition, with more than 600 conserved lncRNA m^6^A peaks, such major reorganization was not anymore detectable within the later stages when P7 and P28 were compared, suggesting that the methylome remains highly redundant but it was shrinking with an age increase (Fig. 2A–C). Notably, we found a major m^6^A peak at a rough locus of 50–55kB within chromosome 14 at P1, which disappeared at P7 and P28 (Fig. 2F). Through the analysis of the data, the decrease of the m^6^A level of lncRNA *Myh6* and lncRNA *Myh7* may be responsible for the disappearance of this single m^6^A peak (Supplemental Table S5). *Myh6* was primarily expressed in the cardiomyocytes in adult mice but *Myh7* was expressed in embryonic cardiomyocytes [37]. Hypertrophy of adult hearts is also associated with *Myh6* downregulation and *Myh7* induction, returning to a fetal state of MHC expression and thus controlling the expression of MHC may be an attractive approach for heart failure therapy [58]. It has been reported that inhibition of thyroid hormone activaty prolonged myocardium regenerative ability [13], but the role of myosin isoforms, especially from epitranscriptomic m^6^A, in this process has not been established yet. Whether m^6^A modification is involved in the shift from *Myh6* to neonatal *Myh7* expression needs to be studied further. Taking these above observations into account, during the period of rapid changes of myocardial regeneration ability (P1 to P7), m^6^A may exert potential influence within the loss of myocardial regenerative capacity after birth.

Subsequently, through combined analysis of the m^6^A-Seq and RNA-seq data, it revealed the correlation between m^6^A modification and lncRNA expression level in the developing mouse hearts(P1, P7 and P28). We explored that in P7 vs P1, m^6^A modification was associated with lncRNA expression levels to some extent (Fig. 3A). However, no association was seen between m^6^A modification and lncRNA expression level in P28 vs P1 and P7 vs P28 (Fig. 3B, C). We speculate that m^6^A modifications may affect the expression of certain lncRNAs in the heart from P1 to P7, and that this effect was diminished after P7. Interestingly, our previous study found that the expression of m^6^A reader, insulin-like growth factor-binding protein 3(IGF2BP3), was decreased gradually after birth [59], and its overexpression was also reported to enhance cardiomyocyte proliferation both in vitro and in vivo [49]. Furthermore, it is very likely that lncRNAs were differently expressed due to differential m6A methylation. Indeed, we found that lncRNA *Snhg3* was downregulated while lncRNA *Nedd4* was upregulated due to m6A.

We further identified 38 lncRNAs with different m^6^A modification and lncRNA expression levels in the P7 vs P1 (Fig. 4A, Supplemental Table S6) and 9 of these 38 (Supplemental Table S1) may be potentially related to cardiac development or regeneration or may be the new molecules that potentially regulates myocardial regeneration. After removing lncRNA *Ece1*, which was not match with the expression change in P1 to P7 of RNA-sequence (Fig. 4B), we performed knock-down METTL3 in neonatal mouse cardiomyocytes to verify the expression of the remaining 8 lncRNAs, there were 6 lncRNAs having notable changes in the expression level (Fig. 4C, D). We chose the lncRNA with the largest increase in expression (lncRNA *Snhg3*) and the lncRNA with the largest decrease in expression (lncRNA *Nedd4*) after knockdown of METTL3 to conduct MeRIP-qpcr, and the results of MeRIP-qpcr data showed that the m^6^A abundances of ‘lncRNA *Snhg3*’ and ‘ lncRNA *Nedd4*’ were also decreased (Fig. 4E, F). This interesting finding suggests that m^6^A modification may indeed affect the expression of the key lncRNAs involved in the cardiac development but the potential underlying role of m^6^A readers executing such divergent responses still need further study. Recently, ubiquitination as an important protein post-translational modification has been demonstrated to be closely related to cardiovascular disease [60], and Nedd4 as the key enzyme in ubiquitination has been found to be involved in the regulation of myocardial regeneration and repair [31]. According to a recent study, USP12 (ubiquitin-specific protease 12) via enhancing p300/MTLL3 axis promote myocardial hypertrophy [61], and our finding may indicate the possibility of m^6^A methylation and ubiquitination cooperatively regulate heart growth. We also found that *lncGm15328* and lncRNA *Zfp597* may partake the loss of myocardial regeneration ability during mouse heart development from P1 to P7 (Fig. 5), and that m^6^A modification may also be involved in this process by regulating the expression levels of these two lncRNAs. The function of these two lncRNAs in heart growth has not been reported and may have great potential as the new targets for regulating myocardial regeneration, but warrants further study with MI models.

Additionally, GO and KEGG analysis revealed that both the hypermethylated and the hypomethylated lncRNAs (P7 vs P1 and P28 vs P1 group) were involved in many important biological functions and pathways. For instance, the GO results showed that hyper/hypomethylated lncRNAs may enrich in ‘regulation of organ growth’, ‘muscle organ development’ and ‘response to thyroid hormone’ functions. It was reported that the loss of heart regenerative capacity was triggered by increasing thyroid hormones [13]. In addition, the analysis indicated that some m^6^A differential lncRNAs were also enriched in ‘insulin-like growth factor receptor’ and ‘response to angiotensin’, which are participating in cardiac hypertrophy [36, 44]. m^6^A differential lncRNAs enriched in myocardial regeneration-related pathways, such as ‘TGF-beta signaling pathway’ is well documented [62]. All the above indicates that m^6^A differential lncRNAs may play an important role in the heart normal or dysfunction development.

In conclusion, we have provided an overall framework for m^6^A-lncRNA-heart development, and our data provides stagewise views into early postnatal myocardial N^6^-methyladenosine (m^6^A) methylomes specifically in long non-coding RNAs (lncRNAs), which, when correlated with the known simultaneously occurring loss of inherent cardiac regenerative capacity, were appropriately pointed out to be of interest regarding possible functional roles with the cardiac regeneration capacity. These findings identify potential m^6^A methylated lncRNA targets for future experimental heart regeneration-targeted studies.

## Materials and methods

### Animals studies

The animal study was reviewed and approved by the institutional committee of animal care and use of the Affiliated First Hospital of Guangdong Medical University (Guangdong, China). C57BL/6 mice, Male, aged 1-day-old, 7-day-old and 28-day-old, were purchased from the Experimental Animal Center of Southern Medical University (Guangdong, China). Under the premise of meeting the inclusion criteria, we randomly selected mice from different groups for heart extraction and sequencing. We assigned three groups in line with different ages (P1, P7, and P28) and collected three biological replicates (*n* = 3–5 mice per group) among which none was excluded. There was no blind selection involved. All mice in both groups were appropriately anesthetized with Ketamine (80 mg/kg, IP) + Xylazine (10 mg/kg, IP) and sacrificed by cervical dislocation. Their cardiac tissue was immediately collected, frozen in liquid nitrogen and stored at −80 °C for later preparation of total RNA.

### Total RNA preparation

Total RNA was harvested and extracted from tissue samples using Trizol Reagent (Thermo Fisher Scientific, MA, USA). The accurate concentration and sample purity were detected through NanoDropND-2000 (Thermo Fisher Scientific, MA, USA). Finally, the degradation of total RNA was determined by agarose gel electrophoresis and Agilent 2100 Bioanalyzer (Agilent Technologies Inc, CA, USA). Only RIN (RNA integrity number)>7 of extracted RNA was used to ensure downstream high-quality total RNA-seq library construction **(**Supplemental Table S7**)**. Qualified RNA acquisition carried out the above series of quality control (QC) processes.

### lncRNAs Library Construction and sequencing

In brief, according to the manufacturer’s instructions, the total RNA was used for removing the rRNAs with Ribo-off rRNA Depletion Kit (H/M/R) (Vazyme Biotech, China) and purified by AMPure XP magnetic beads (Beckman Coulter, CA, USA). ABI 2720 Thermal Cycler (Thermo Fisher Scientific, USA) was used to construct RNA libraries using fragmented rRNA-depleted RNAs. The library concentration was accurately quantified by Qubit and the size distribution of library fragment was determined by Agilent 2100 Bioanalyzer (Agilent Technologies Inc, CA, USA). Then, the libraries were captured on Illumina cbot Cluster Station (Illumina, CA, USA) and finally sequenced and visualized for corresponding cycles on Illumina Hiseq 2500 (Illumina, CA, USA).

### lncRNAs methylation-RNA immunoprecipitation (MeRIP) library construction and sequencing

MeRIP-Seq was based on previously published procedures [28]. Briefly, fragmented RNA was incubated with immunomagnetic beads premixed anti-m^6^A antibody. The mixture was then immunoprecipitated by incubation with protein-A beads. Next, purified RNA was used for the RNA-seq library by Illumina Hiseq 2500 (Illumina, CA, USA). The input sample without immunoprecipitation and the m^6^A IP samples were subjected to PE150 paired-end sequencing InIllumina Novaseq™ 6000. After removal of ribosomal sequences, the percentage of data quality values greater than Q30 was more than 90%. Finally, methylated sites on RNAs (peaks) were identified by the ChiPseeker package [63].

### Sequencing Data Analysis

To identify lncRNAs, the reported databases and software (EggNOG, CNCI, Pfam, CPC2) were applied based on the noncoding potential property of lncRNAs. The up- or downregulated expression of lncRNAs was set at absolute fold change (FC > 1.5, and *P* < 0.05). For m^6^A sequencing, methylated sites on lncRNAs (m^6^A peaks) were identified with the diffReps differential analysis package [64] with differential fold change evaluated of > 1.5 and a *P* value < 0.05. The correlation analysis between the lncRNAs (lncRNAs with changes in m^6^A modification) and co-expression mRNAs was evaluated using Pearson correlation by SPSS software (v22.0)

### GO and KEGG Pathway Databases Analysis

LncRNAs expression with differentially methylated profiles were compared to functional differences between-group variance using enrichment analysis. Gene ontology (GO) was performed to annotate these genes. The functions were distinguished into three parts: cellular component (CC), molecular function (MF), and biological process (BP). The *p* value denotes the significance of GO term enrichment of the genes. In addition, Pathway enrichment analysis is a functional analysis that maps genes to the Kyoto Encyclopedia of Genes and Genomes (KEGG). The Fisher *p*-value denotes the significance of the pathway correlated to the conditions [65, 66].

### Cardiomyocyte Isolation and Culture

Neonatal cardiomyocytes were isolated from 1 day-old (P1) and 7-day-old (P7) C57BL/6 mice by enzymatic dissociation. Shortly, P1 or P7 hearts were rapidly obtained and removed mostly blood in ice-cold PBS (C10010500BT, Gibco, USA) before being digested with trypsin enzyme (25200056, Gibco, USA). The separated cells were cultured in Gelatin-dealt wells (G8061, Solarbio, China) with 10% FBS DMEM medium (11995-065, Gibco, USA) supplemented with L-glutamine, 1% Antibiotics and 5% CO2 at 37 °C [67].

Transfection of Mettl3 siRNA (5′-GCUACCGUAUGGGCACUUATT-3′)/scrambled controls (RiboBio, Guangzhou, China) in cardiomyocytes was using Lipofectamine RNAiMAX reagent (Invitrogen, CA, USA) following the manufacturer’s instructions. Cardiomyocytes from neonatal heart were transfected with si-NC or si-METTL3 for 72 h, then the expression of METTLE3 and lncRNAs were determined.

### Quantitative Real-time PCR

Total RNA isolation from tissue and the cultured primary cardiomyocytes were extracted with Trizol Reagent (Thermo Fisher Scientific, MA, USA). The cardiomyocyte lncRNA were measured by RT-qPCR. The ratio of OD260/280 to OD260/230 of the extracted RNA were detected by NanoDrop (Thermo Fisher Scientific, Waltham, MA, USA) as the samples purity index. If the OD260/280 value was between 1.8 and 2.2, OD260/230 ≥ 2.0, the RNA purity and integrity were suitable for downstream experiments. Quantitative reverse transcription-polymerase chain reaction (qRT-PCR) was performed on LightCycler 480 System (Roche, Germany) or Applied Biosystem 7500 qPCR system (Applied Biosystems,USA) by using SYBR Green PCR Master Mix (Thermo Fisher Scientific, MA, USA) and the prepared cDNAs.

### Western blot analysis

Total protein was extracted with RIPA lysis buffer, and the protein concentrations were determined by enhanced bicinchoninic acid (BCA) protein assay kit (Beyotime, Shanghai, China). Furthermore, the equivalent amounts of protein were separated by SDS-PAGE on 10% acrylamide gels and transferred onto polyvinylidene fluoride (PVDF) membranes, blocked with 5% milk, and then incubated with primary target antibodies. The primary antibodies were anti-METTL3 (ab195352, Abcam, UK) and anti-GAPDH (AP0063, Bioword, USA). As the secondary antibody was goat polycolonal anti-Rabbit-IgG (14708 S, CST, USA). Quantitative analysis were performed by Image J software (NIH, Bethesda, USA).

### m^6^A-RIP qPCR

To examine m^6^A modifications on individual genes, the MeRIP m^6^A Kit (GS-ET-001, CouldSeq, China) was used according to the manufacturer’s instructions. RNA in a certain ratio was severed as input control, and further analyzed by qPCR with the primers (Supplemental Table S1). The related enrichment of m^6^A in goal genes was calculated by normalizing the value of amplification cycle to the corresponding input portion.

### Constructing the ceRNA network

The lncRNA-miRNA-mRNA co-expression network was visualized with the Cytoscape software (http://www.cytoscape.org/). Using the miRDB (Version5.0; http://mirdb.org) to predict the miRNAs targeted by lncRNAs. mRNAs targeted by the miRNAs were retrieved from the miRDB (Version 5.0; http://mirdb.org), miRTarBase (Version7.0; http://mirtarbase.mbc.nctu.edu.tw/), and TargetScan (Version 7.2; http://www.targetscan.org/vert_72/) databases [68–70].

### Statistical analysis

All data were expressed as the mean ± SEM (standard error mean) and analyzed with Student t-test or hypergeometric test (Prism 9.0, GraphPad Software Inc, San Diego, CA). A P value less than 0.05 was considered to be of statistical significance.

## Supplementary information

SUPPLEMENTAL MATERIAL

The first western blot

The first western blot

The first western blot

The first western blot

The first western blot

The first western blot

The second western blot

The second western blot

The second western blot

The second western blot

The second western blot

The second western blot

The third western blot

The third western blot

The third western blot

The third western blot

The third western blot

The third western blot

## Data Availability

All computer codes were used to generate results during this study are available from the corresponding authors on reasonable request.

## Data availability

The datasets generated and analyzed during the current study are available in the NCBI (https://www.ncbi.nlm.nih.gov/home/download/) repository, and BioProject ID: PRJNA828395.
