# Supplementary material for: N6-methyladenosine modulates long non-coding RNA in the developing mouse heart
Source: Cell Death Discov. 2022 Jul 20;8:329. doi: 10.1038/s41420-022-01118-x (PMC9300643; doi:10.1038/s41420-022-01118-x)
Supplement: Supplementary file 1 — SUPPLEMENTAL MATERIAL [file 41420_2022_1118_MOESM1_ESM.doc]

**Supplemental Tables**

***Table S1*. The primers used for qPCR in this study**

| **Primers name** |  | **Sequence (5'-3')** | **Reference** |
| --- | --- | --- | --- |
| **Dnaja3(ENSMUST00000144815)** | **F** | **CACCTGAATGACAGCCAAGGTT** | **doi: 10.1038/nm1327** |
|  | **R** | **CCCAGCAGAACTTGAAACACTC** |  |
| **Ece1(ENSMUST00000129607)** | **F** | **GTCCAAGAGCAAATTGAAGCTC** | **doi: 10.1172/JCI7447** |
|  | **R** | **TCCACGACAACTTCTGATCCTG** |  |
| **Snhg3(ENSMUST00000141090)** | **F** | **TGTGGACCATGACTGAGGAGAC** | **doi:10.1007/s13577-019-00287-9** |
|  | **R** | **ATCATTGACCGGGTCTGCAA** |  |
| **Gprasp2(ENSMUST00000141953)** | **F** | **AGAGAACACCCAGGAATAGGAGTC** | **doi: 10.1084/jem.20150806** |
|  | **R** | **AGCCTTAAAGCAGGGCTGGC** |  |
| **Ccnd3(ENSMUST00000182060)** | **F** | **CATGGATGTCAATCATTCCTGTCA** | **doi: 10.1139/y2012-037** |
|  | **R** | **AAGGTCTTGCTGGTCCATAGCTT** |  |
| **Zeb2(ENSMUST00000238346)** | **F** | **GGATTGCGAGTTTGCACGT** | **doi:10.1038/s41467-020-20361-3** |
|  | **R** | **CGAGCAAAGGCTGATGATACC** |  |
| **Nedd4(ENSMUST00000184386)** | **F** | **AGTACTCTCGGAGGACGAGTGA** | **doi:10.1161/CIRCULATIONAHA.118.038361** |
|  | **R** | **AGTTGTTCCTCTGTAACAGACGGTT** |  |
| **Sema4d(ENSMUST00000155896)** | **F** | **CTAAAGTGGCTTCTGTGGTAGGAC** | **doi:10.1371/journal.pone.0064265** |
|  | **R** | **ACGACGTCATGCCAAGGCT** |  |
| **Firre(ENSMUST00000204729)** | **F** | **ATTAGGCTCAGAGCTGCTTGGTC** | **doi: 10.1016/j.bbrc.2019.01.105** |
|  | **R** | **TAACACCACGGACGGGCTTT** |  |

***Table S2*. Top ten up-methylation peaks (P7 vs P1)**

| **Chr** | **TxStart** | **TxEnd** | **lncRNA** | **Biotype** | **Fold Change** |
| --- | --- | --- | --- | --- | --- |
| 14 | 54973303 | 54973352 | **Myh7** | Retained intron | 4.7835 |
| 8 | 106010920 | 106010995 | **Dus2** | Retained intron | 4.5052 |
| 4 | 137862261 | 137905124 | **Ece1** | Processed transcript | 4.3816 |
| 9 | 119065340 | 119065580 | **Vill** | Processed transcript | 4.3131 |
| 15 | 101352223 | 101356940 | **Krt80** | Retained intron | 3.6388 |
| 14 | 54973161 | 54973341 | **Myh7** | Retained intron | 3.2766 |
| 18 | 16821262 | 16821312 | **LncGm15328** | lncRNA | 3.2327 |
| 8 | 57507665 | 57507740 | **Hmgb2** | Processed transcript | 2.9341 |
| 1 | 171338240 | 171338415 | **Dedd** | Retained intron | 2.8686 |
| 9 | 119063581 | 119063631 | **Vil** | Processed transcript | 2.8397 |

Top ten up-methylation peaks and their corresponding lncRNAs (P7 vs P1; FC >1.5, *P* < 0.05); P <0.05 (n =3) (Student t-test).

***Table S3*. Top ten down-methylation peaks (P7 vs P1)**

| **Chr** | **TxStart** | **TxEnd** | **lncRNA** | **Biotype** | **Fold Change** |
| --- | --- | --- | --- | --- | --- |
| 2 | 77503663 | 77519458 | **Zfp385b** | Processed transcript | 23.9389 |
| 18 | 6790079 | 6790154 | **Rab18** | Processed transcript | 12.8930 |
| 1 | 137953246 | 137953321 | **Ptprc** | Retained intron | 11.5971 |
| 2 | 48934075 | 48934125 | **Orc4** | Processed transcript | 11.2318 |
| 16 | 3865091 | 3865141 | **Zfp597** | Processed transcript | 11.2224 |
| 3 | 60624877 | 60624952 | **Mbnl1** | Retained intron | 7.8527 |
| 13 | 28419887 | 28419987 | **Gm6081** | lncRNA | 7.5153 |
| 2 | 45105386 | 45105436 | **Zeb2** | Processed transcript | 7.1529 |
| 15 | 73394512 | 73394562 | **Ptk2** | Retained intron | 6.4659 |
| 5 | 130135615 | 130135729 | **Tpst1** | Retained intron | 6.1898 |

Top ten down-methylation peaks and their corresponding lncRNAs (P7 vs P1; FC >1.5, *P* < 0.05); P <0.05 (n =3) (Student t-test).

***Table S4.* Top ten up-methylation peaks (P28 vs P1)**

| **Chr** | **TxStart** | **TxEnd** | **lncRNA** | **Biotype** | **Fold Change** |
| --- | --- | --- | --- | --- | --- |
| 16 | 3865066 | 3865141 | **Zfp597** | Processed transcript | 15.0325 |
| 7 | 38195444 | 38195519 | **1600014C10Rik** | Processed transcript | 11.0946 |
| 1 | 133301680 | 133301805 | **Plekha6** | Retained intron | 10.6500 |
| 7 | 93000471 | 93000621 | **Gm31663** | Retained intron | 8.2745 |
| 1 | 180179183 | 180179573 | **Coq8a** | Retained intron | 7.8486 |
| 1 | 9558222 | 9558272 | **Adhfe1** | Nonsense | 7.7204 |
| 19 | 9830398 | 9830673 | **Gm50321** | lncRNA | 7.3995 |
| 1 | 178481562 | 178481637 | **Efcab2** | Processed transcript | 7.0143 |
| X | 133582859 | 133582984 | **Pcdh19** | Processed transcript | 6.7446 |
| 3 | 60528313 | 60528438 | **Mbnl1** | Retained intron | 6.5616 |

Top ten up-methylation peaks and their corresponding lncRNAs (P28 vs P1; FC >1.5, *P* < 0.05); P <0.05 (n =3) (Student t-test).

***Table S5 .* Top ten down-methylation peaks (P28 vs P1)**

| **Chr** | **TxStart** | **TxEnd** | **lncRNA** | **Biotype** | **Fold Change** |
| --- | --- | --- | --- | --- | --- |
| 14 | 54953836 | 54953886 | **Myh6** | Retained intron | 64.5459 |
| 14 | 54984722 | 54984772 | **Myh7** | Retained intron | 21.0698 |
| 1 | 57857494 | 57879635 | **Spats2l** | Retained intron | 12.3979 |
| 19 | 5082119 | 5082269 | **Tmem151a** | Retained intron | 6.4266 |
| 9 | 96437657 | 96438007 | **BC043934** | lncRNA | 5.6388 |
| 4 | 134468319 | 134469781 | **Stmn1** | Processed transcript | 5.2104 |
| 7 | 68186907 | 68186982 | **Igf1r** | Retained intron | 4.0371 |
| 9 | 69467310 | 69467360 | **Anxa2** | Retained intron | 3.8505 |
| 4 | 137905099 | 137905149 | **Ece1** | Processed transcript | 3.2003 |
| 9 | 69479614 | 69479664 | **Anxa2** | Retained intron | 3.0723 |

Top ten down-methylation peaks and their corresponding lncRNAs (P28 vs P1; FC >1.5, *P* < 0.05); P <0.05 (n =3) (Student t-test).

***Table S6.* The list of 38 lncRNAs that exhibit changes in both m6A level and lncRNAs expression in P7 vs P1 mouse heart tissue (FC >1.5, *P* < 0.05); P <0.05 (n=3)(Student t-test).**

| **Gene name** | **Pattern** | **Chromosome** | **m^6^A level change** | | | **lncRNA level change** |
| --- | --- | --- | --- | --- | --- | --- |
|  |  |  | **TxStart** | **TxEnd** | **Fold Change** | **Fold Change** |
| **Gm26885** | hyper-down | 17 | 29491357 | 29491482 | 1.8123 ↑ | 72.2171 ↓ |
| **Dnaja3** | hyper-down | 16 | 4647423 | 4647523 | 2.1510 ↑ | 11.1449 ↓ |
| **LncGm15328** | hyper-down | 18 | 16821262 | 16821312 | 3.2327 ↑ | 159.821 ↓ |
| **Ece1** | hyper-down | 4 | 137862261 | 137905124 | 4.3816 ↑ | 2.7790 ↓ |
| **Bclaf1** | hypo-down | 10 | 20318276 | 20318351 | 3.1559 ↓ | 5.2260 ↓ |
| **Prpf4b** | hypo-down | 13 | 34901834 | 34901959 | 1.9248 ↓ | 37.3835 ↓ |
| **Efr3a-207** | hypo-down | 15 | 65787183 | 65787851 | 1.5035 ↓ | 496.4172 ↓ |
| **Snhg3** | hypo-down | 4 | 132352687 | 132352737 | 1.9785 ↓ | 4.2363 ↓ |
| **Gprasp2** | hypo-down | X | 135887344 | 135887394 | 1.6564 ↓ | 12.2101 ↓ |
| **Ccnd3** | hypo-down | 17 | 47597432 | 47597607 | 1.7982 ↓ | 65.3934 ↓ |
| **Zeb2-230** | hypo-down | 2 | 45105386 | 45105436 | 7.1529 ↓ | 11.1298 ↓ |
| **Nedd4** | hypo-down | 9 | 72662520 | 72662570 | 1.6110 ↓ | 2.6992 ↓ |
| **Zc3h11a** | hypo-down | 1 | 133653983 | 133654108 | 1.6087 ↓ | 35.3717 ↓ |
| **Klhl42** | hypo-down | 6 | 147109904 | 147109954 | 3.0164 ↓ | 193.5477 ↓ |
| **Snhg3** | hypo-down | 4 | 132352687 | 132352737 | 1.9674 ↓ | 54.3992 ↓ |
| **Sema4d** | hypo-down | 13 | 51725271 | 51725371 | 3.0809 ↓ | 23.8763 ↓ |
| **Firre** | hypo-down | X | 50570152 | 50570202 | 1.8333 ↓ | 3.4130 ↓ |
| **Gm26716** | hypo-up | 4 | 130824393 | 130824493 | 1.5101 ↓ | 593.3713 ↑ |
| **Csad** | hypo-up | 15 | 102201059 | 102203157 | 1.9961 ↓ | 161.6199 ↑ |
| **Rb1** | hypo-up | 14 | 73280133 | 73280183 | 1.7813 ↓ | 223.3646 ↑ |
| **Efr3a-204** | hypo-up | 15 | 65787183 | 65787851 | 1.5035 ↓ | 48.7605 ↑ |
| **Neat1** | hypo-up | 19 | 5825757 | 5825857 | 2.6572 ↓ | 11.5183 ↑ |
| **Gm17201** | hypo-up | 10 | 128395448 | 128395563 | 1.6426 ↓ | 27.1416 ↑ |
| **Nktr-215** | hypo-up | 9 | 121754410 | 121754535 | 1.8433 ↓ | 19.2592 ↑ |
| **Hmgxb4** | hypo-up | 8 | 75030726 | 75030876 | 2.1951 ↓ | 3.92782 ↑ |
| **Rbfox2** | hypo-up | 15 | 77097944 | 77099249 | 1.8567 ↓ | 226.3598 ↑ |
| **GRCm38** | hypo-up | 12 | 100209327 | 100209377 | 3.8220 ↓ | 39.0814 ↑ |
| **Mbnl1** | hypo-up | 3 | 60624877 | 60624952 | 7.8527 ↓ | 7.5612 ↑ |
| **Zeb2-217** | hypo-up | 2 | 45105386 | 45105386 | 7.1023↓ | 18.9691 ↑ |
| **Ccnt1** | hypo-up | 15 | 98538688 | 98538763 | 1.6580 ↓ | 520.1254 ↑ |
| **Zfp597** | hypo-up | 16 | 3865091 | 3865141 | 11.2224 ↓ | 383.0060 ↑ |
| **B430212C06Rik** | hypo-up | 18 | 67321233 | 67321458 | 2.2446 ↓ | 65.0834 ↑ |
| **Trim12a** | hypo-up | 7 | 104313902 | 104313952 | 5.6345 ↓ | 47.7476 ↑ |
| **Pign** | hypo-up | 1 | 105658834 | 105659178 | 3.3970 ↓ | 5.7684 ↑ |
| **Gm26690** | hypo-up | 7 | 127984134 | 127984334 | 2.3613 ↓ | 10.1291 ↑ |
| **Mfsd4** | hypo-up | 1 | 132036814 | 132036864 | 1.5908 ↓ | 176.8004 ↑ |
| **Cflar** | hypo-up | 1 | 58714558 | 58714608 | 2.1608↓ | 5.5758 ↑ |
| **Zfp62** | hypo-up | 11 | 49213928 | 49214128 | 2.6809 ↓ | 19.5090 ↑ |

***Table S7. Table with RIN values for each biological replicate.***

| **sample name** | **RNA Concentration(ng/ul)** | **Agilent 2100 RIN** | |
| --- | --- | --- | --- |
| P1(1) | 374 | | 8.30 |
| P1(2) | 335 | | 8.40 |
| P1(3) | 465 | | 8.40 |
| P7(1) | 453 | | 8.40 |
| P7(2) | 218 | | 8.40 |
| P7(3) | 367 | | 8.10 |
| P28(1) | 525 | | 8.70 |
| P28(2) | 729 | | 8.60 |
| P28(3) | 564 | | 8.70 |

**Supplemental Figures**

**
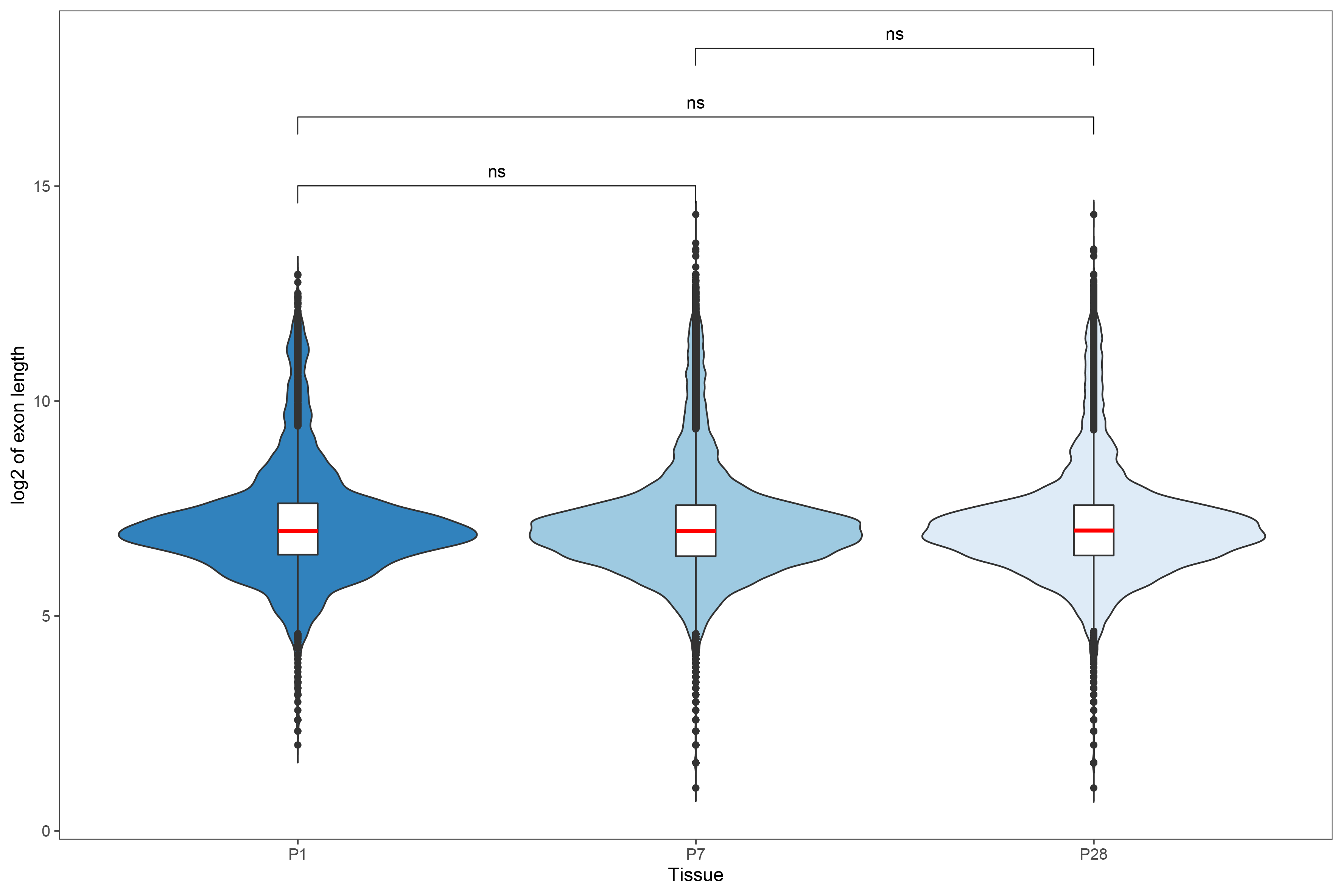
**

**Figure S1**  Exon length of lncRNA with m6A modification in P1, P7 and P28 groups had no statistical difference (n.s.: P >0.05).
