# Supplementary figures and images for: N6-methyladenosine modulates long non-coding RNA in the developing mouse heart
Source: Cell Death Discov. 2022 Jul 20;8:329. doi: 10.1038/s41420-022-01118-x (PMC9300643; doi:10.1038/s41420-022-01118-x)

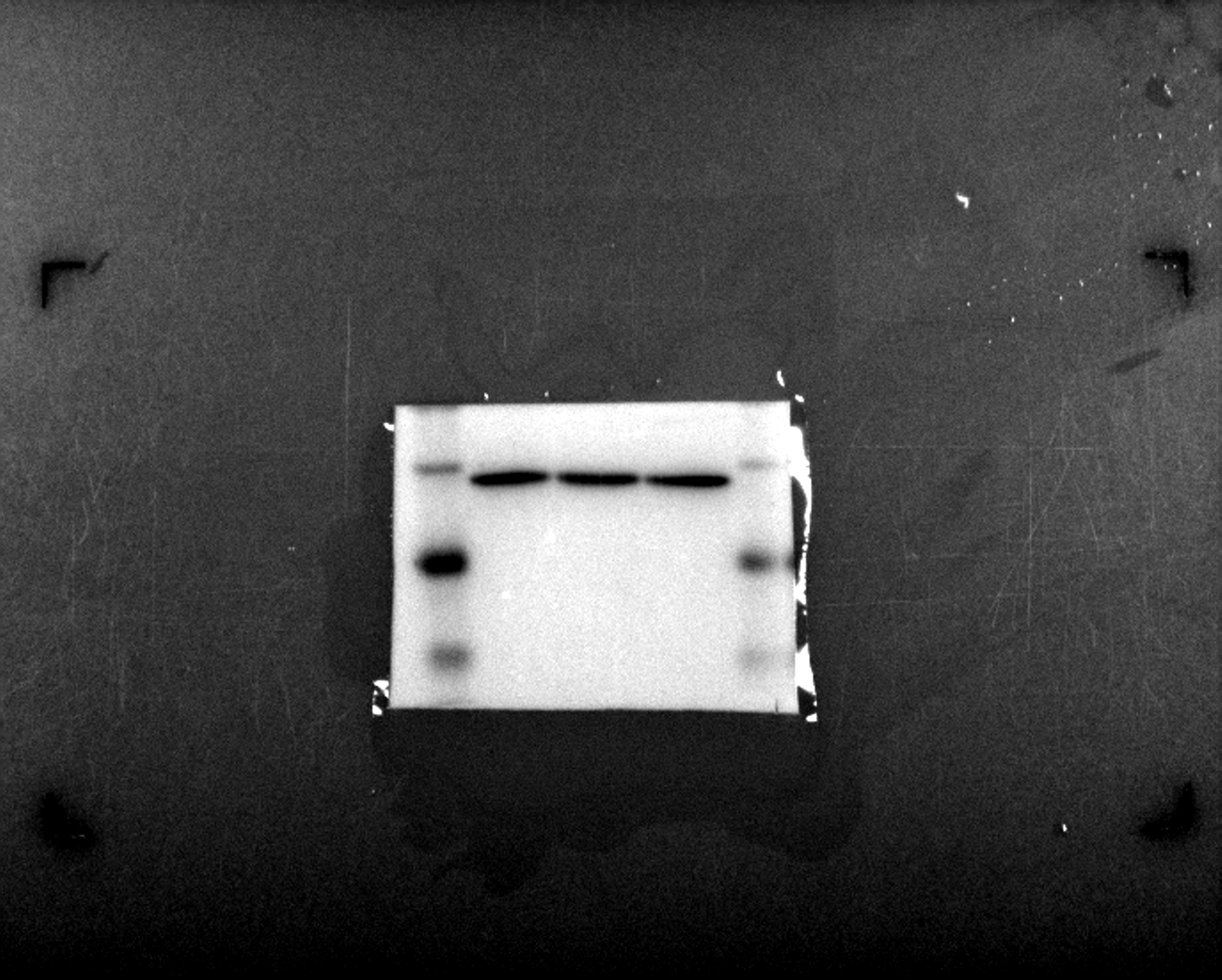

Supplement: Supplementary file 2 — The first western blot [file 41420_2022_1118_MOESM2_ESM.tif]

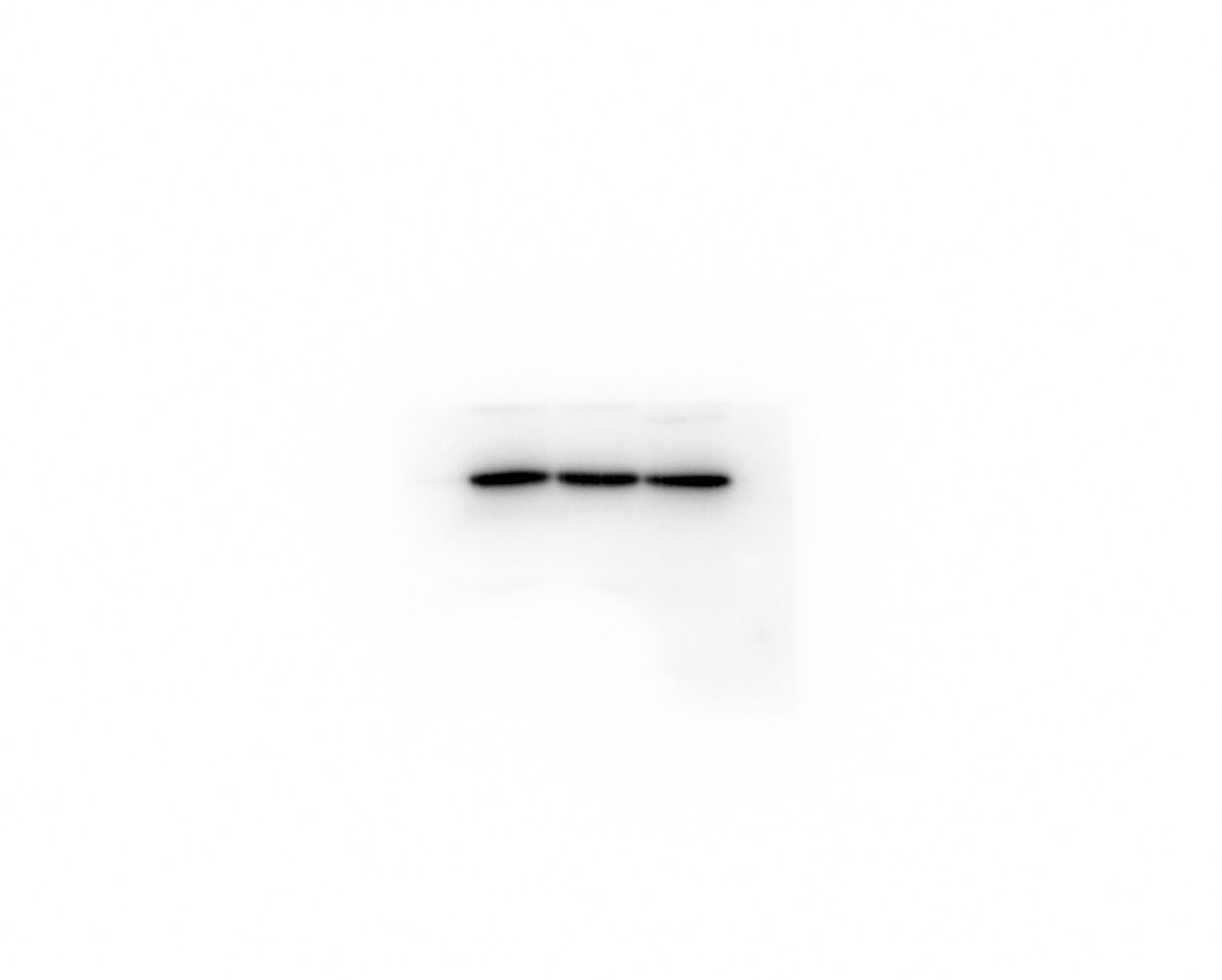

Supplement: Supplementary file 3 — The first western blot [file 41420_2022_1118_MOESM3_ESM.tif]

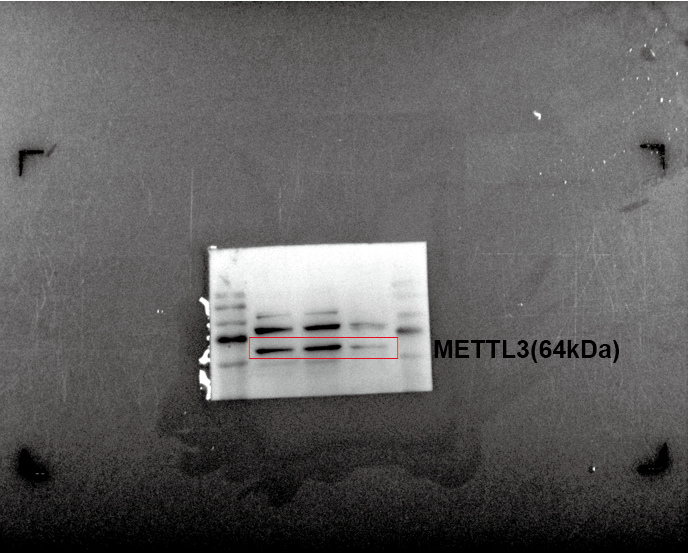

Supplement: Supplementary file 4 — The first western blot [file 41420_2022_1118_MOESM4_ESM.tif]

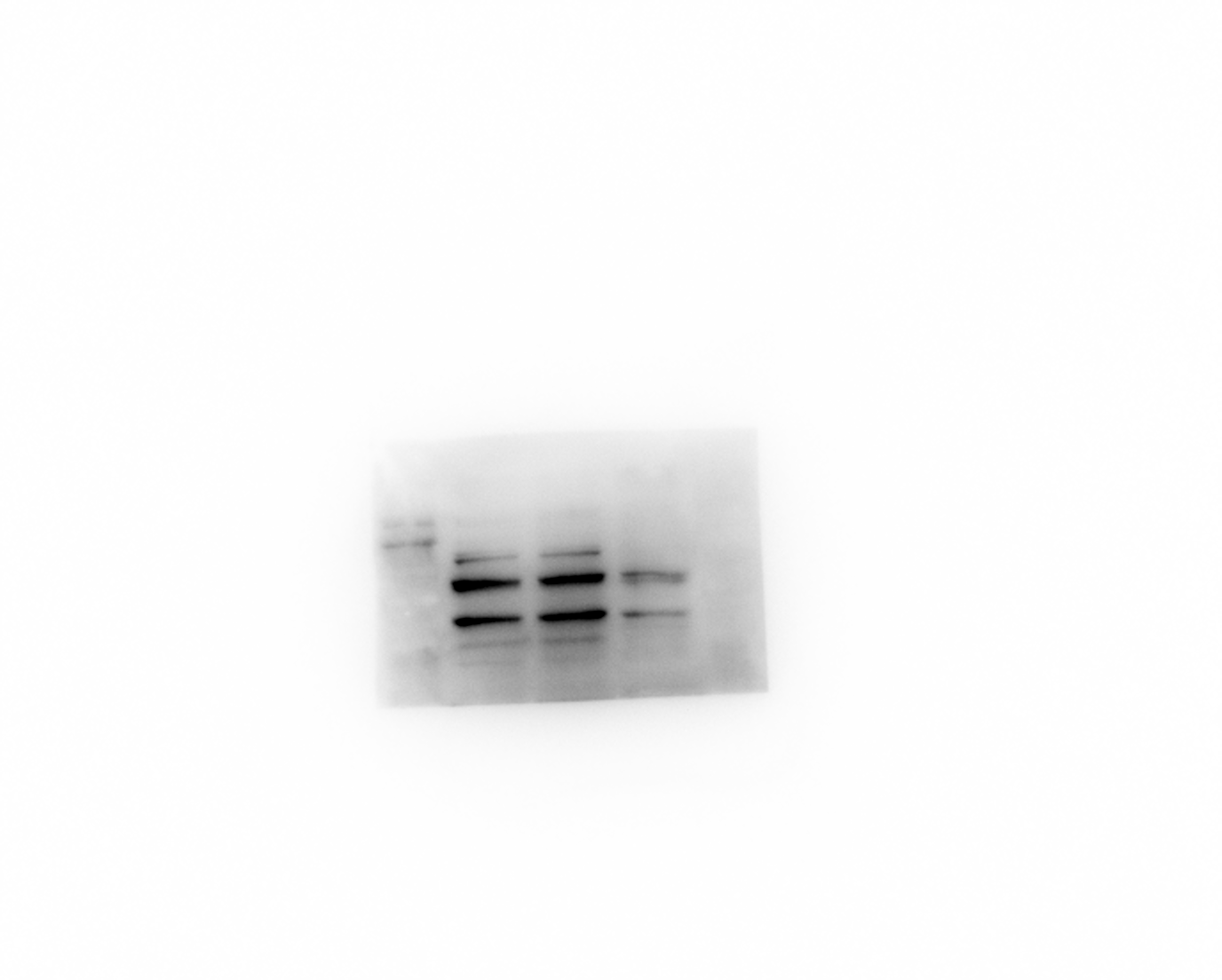

Supplement: Supplementary file 5 — The first western blot [file 41420_2022_1118_MOESM5_ESM.tif]

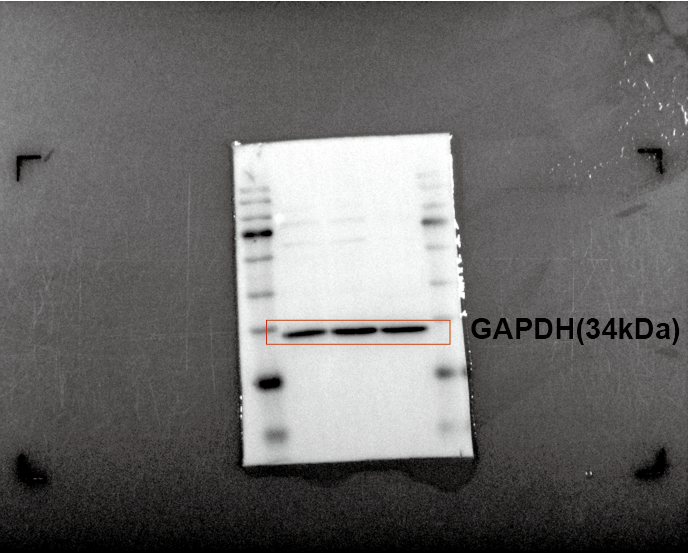

Supplement: Supplementary file 6 — The first western blot [file 41420_2022_1118_MOESM6_ESM.tif]

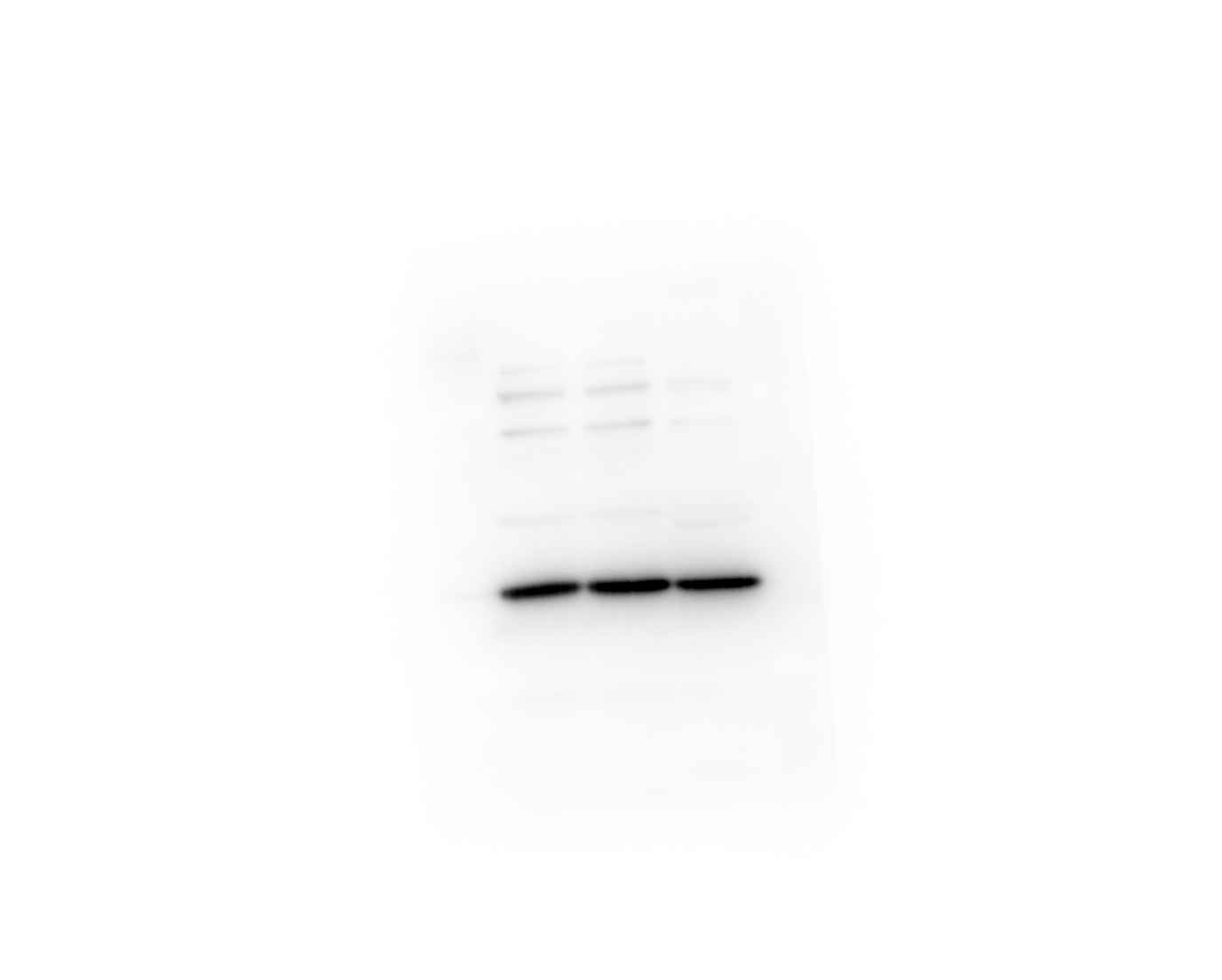

Supplement: Supplementary file 7 — The first western blot [file 41420_2022_1118_MOESM7_ESM.tif]

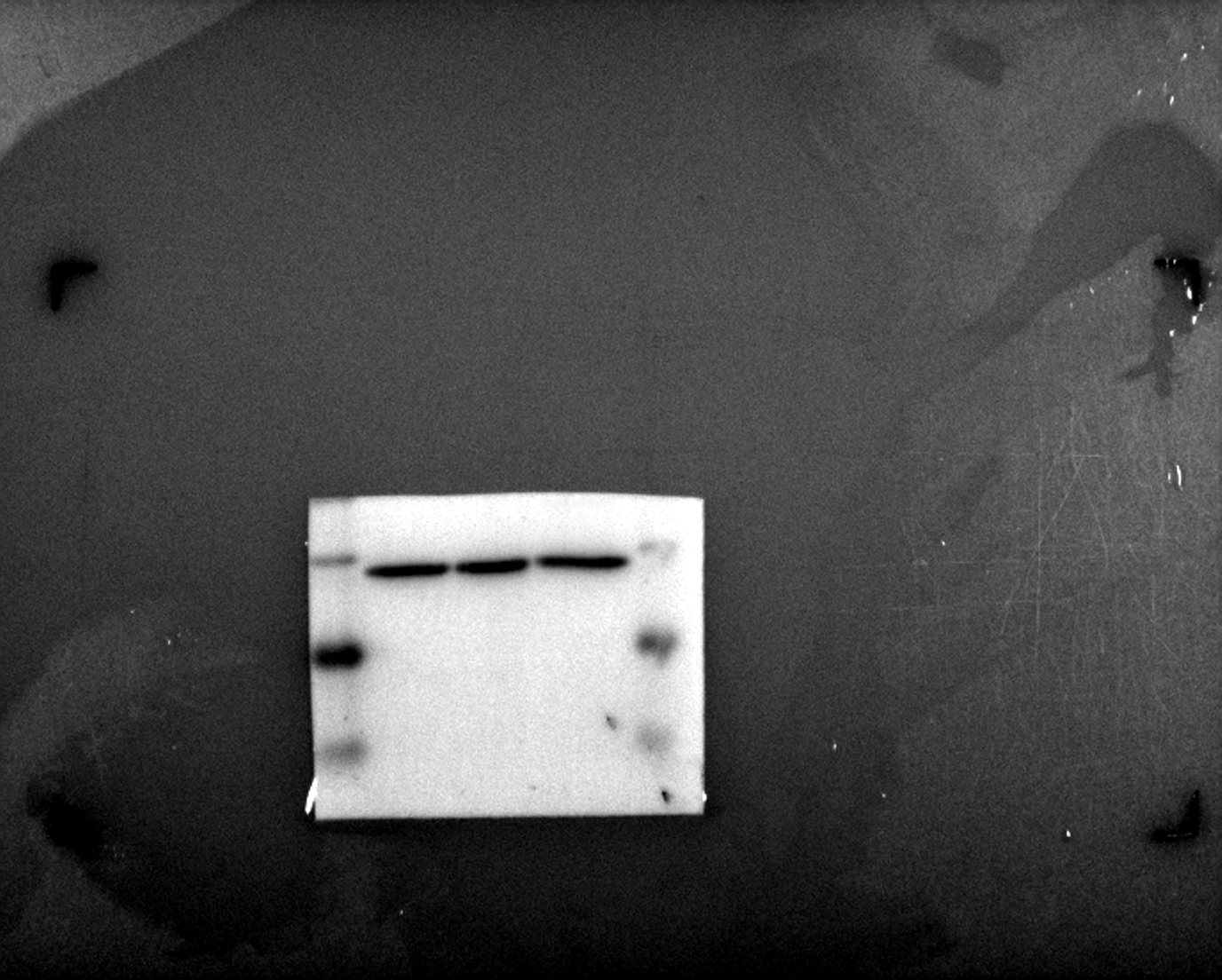

Supplement: Supplementary file 8 — The second western blot [file 41420_2022_1118_MOESM8_ESM.tif]

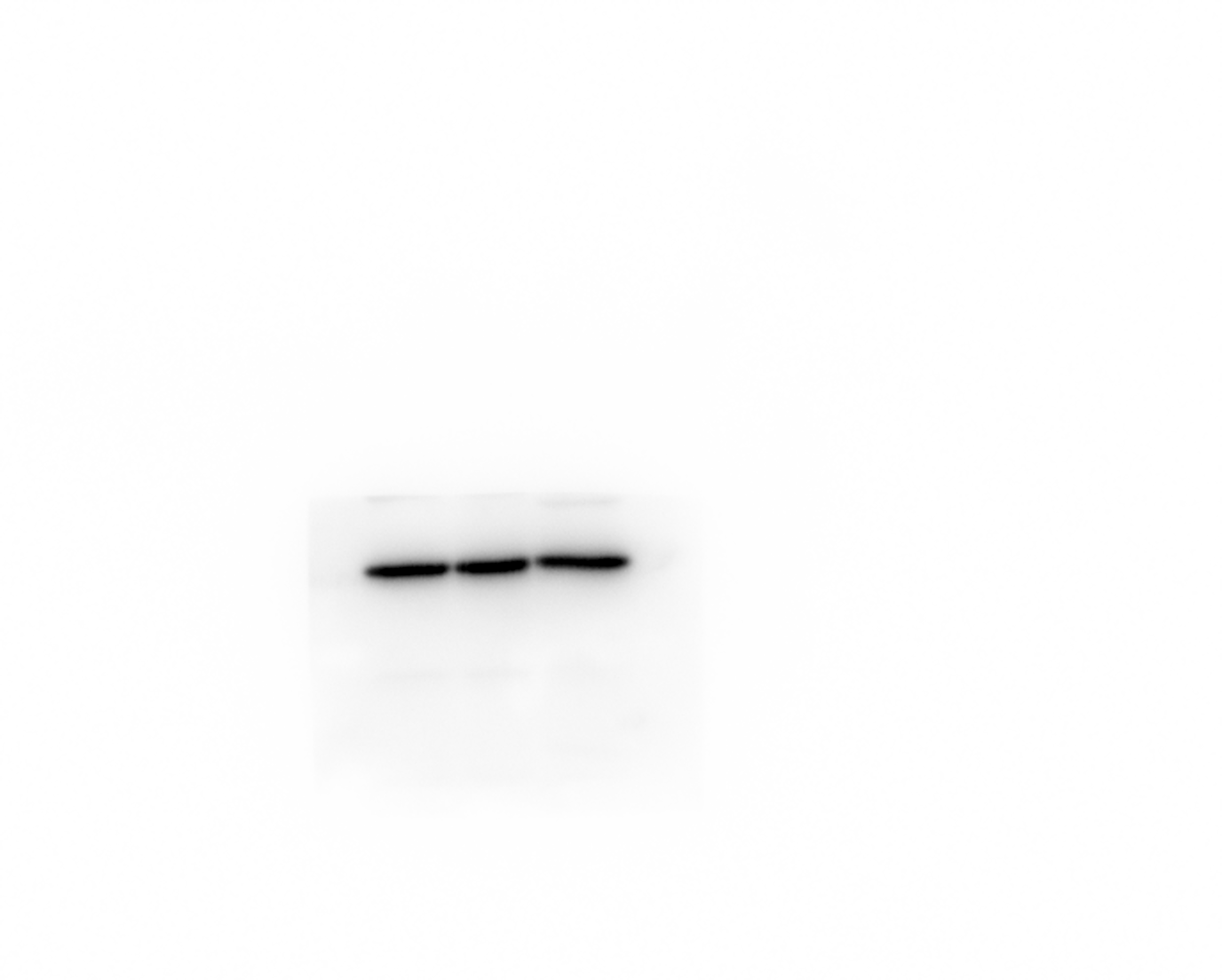

Supplement: Supplementary file 9 — The second western blot [file 41420_2022_1118_MOESM9_ESM.tif]

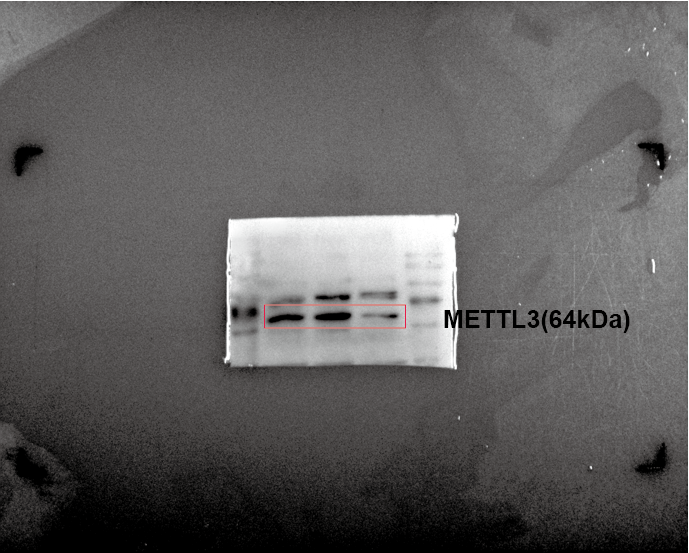

Supplement: Supplementary file 10 — The second western blot [file 41420_2022_1118_MOESM10_ESM.tif]

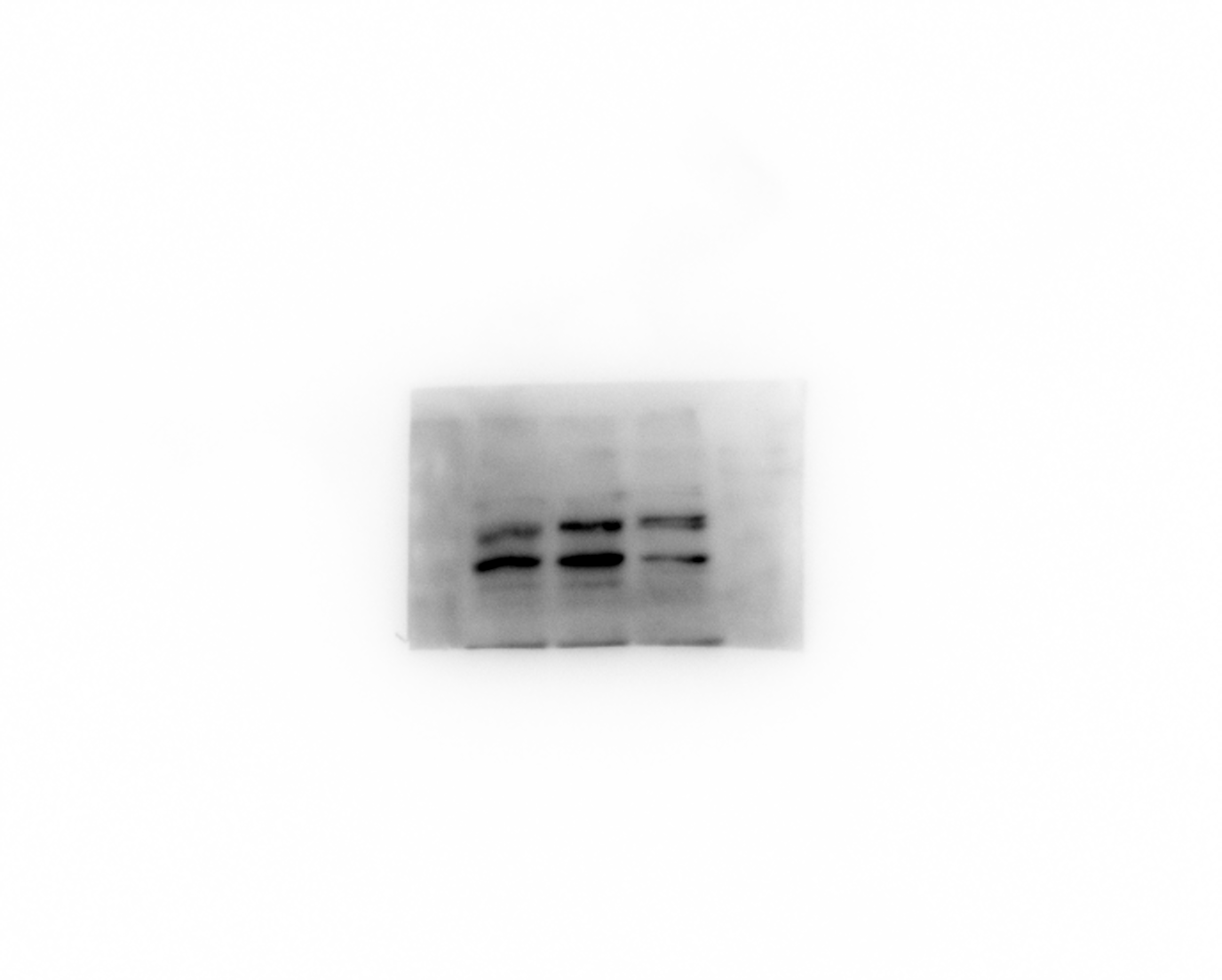

Supplement: Supplementary file 11 — The second western blot [file 41420_2022_1118_MOESM11_ESM.tif]

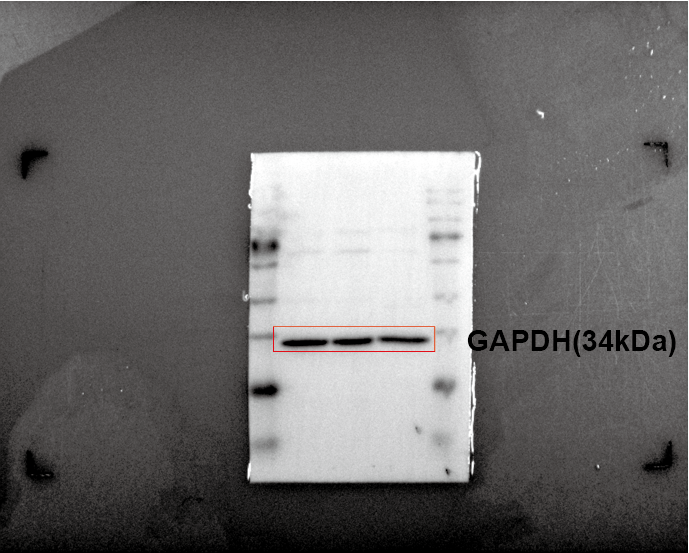

Supplement: Supplementary file 12 — The second western blot [file 41420_2022_1118_MOESM12_ESM.tif]

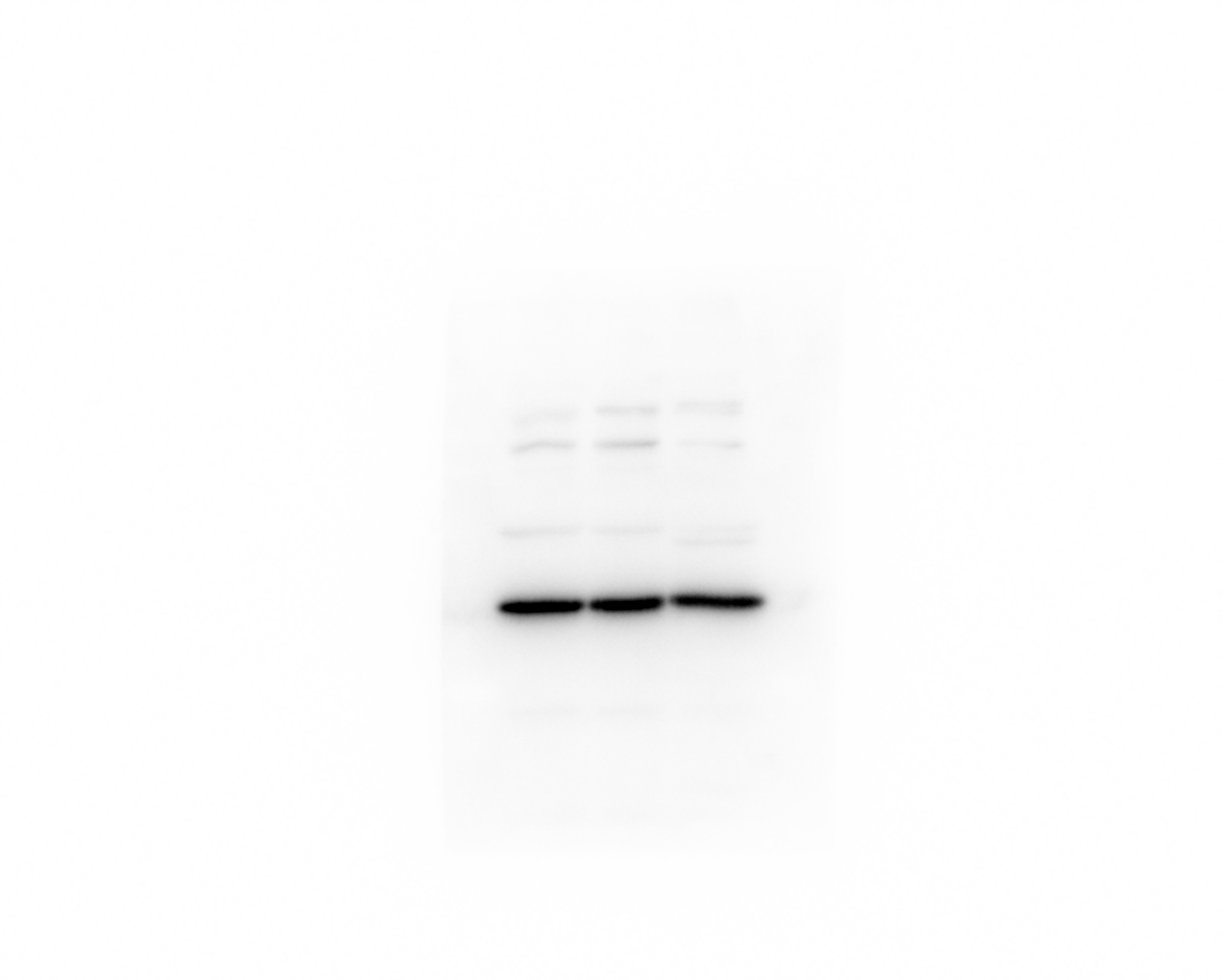

Supplement: Supplementary file 13 — The second western blot [file 41420_2022_1118_MOESM13_ESM.tif]

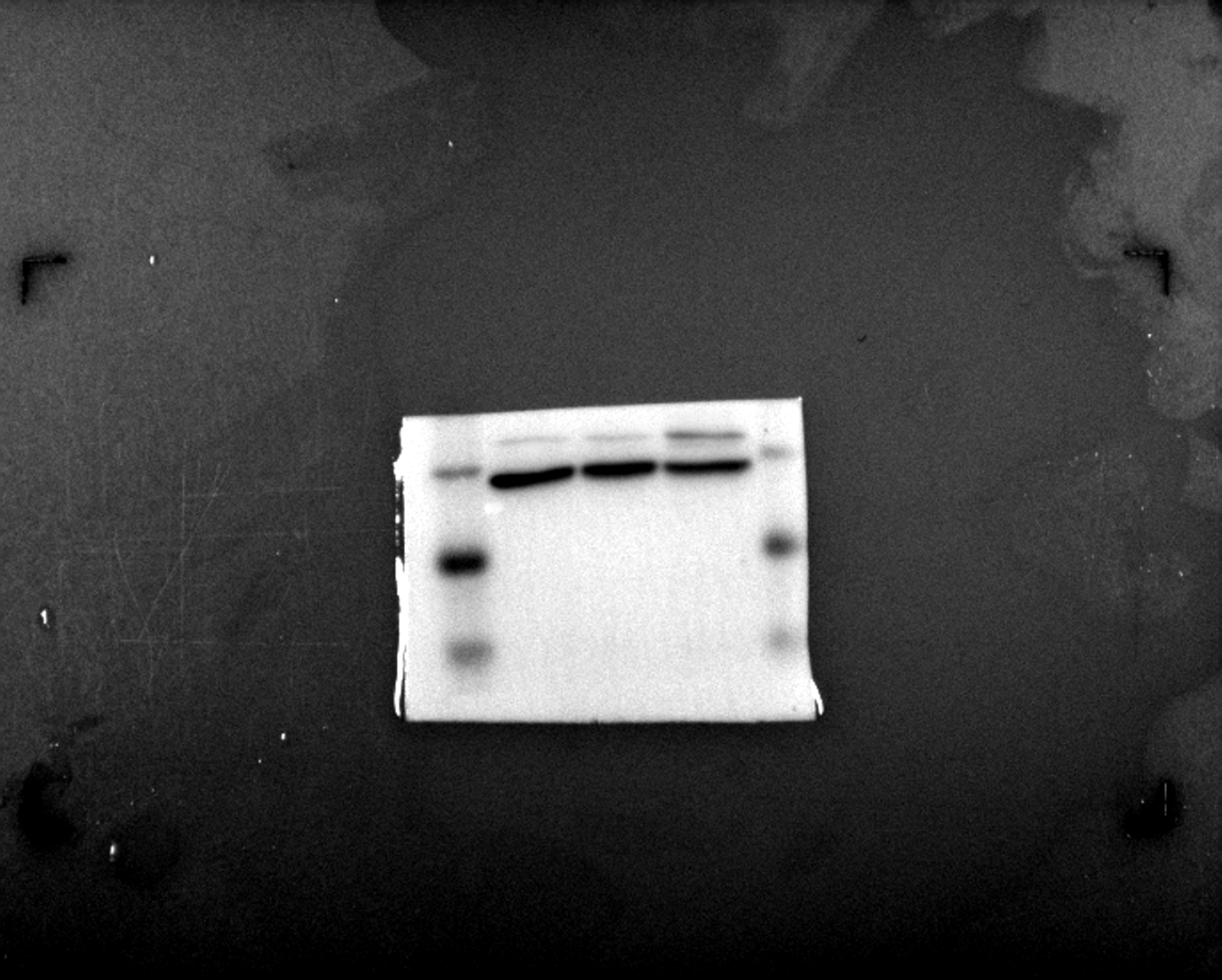

Supplement: Supplementary file 14 — The third western blot [file 41420_2022_1118_MOESM14_ESM.tif]

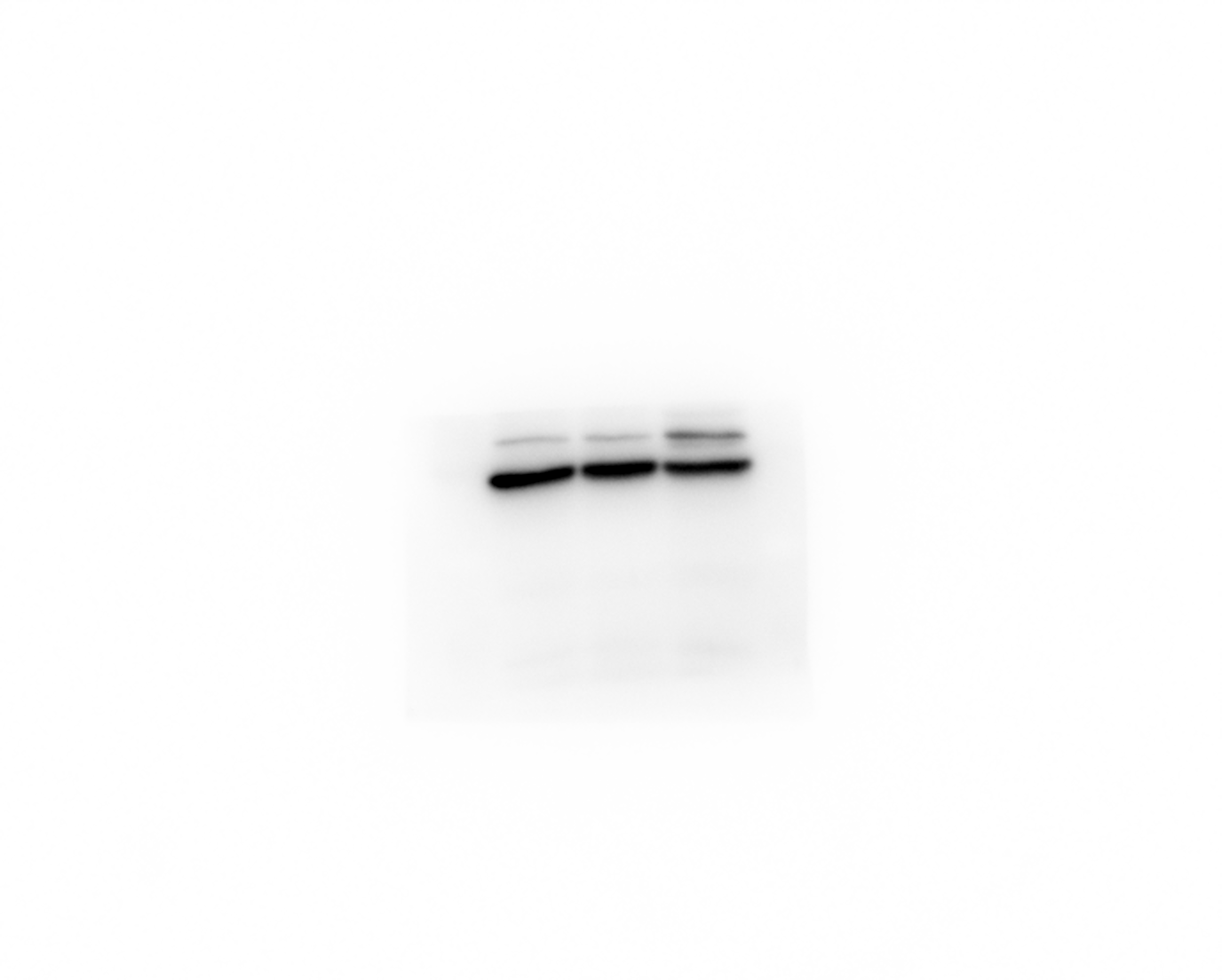

Supplement: Supplementary file 15 — The third western blot [file 41420_2022_1118_MOESM15_ESM.tif]

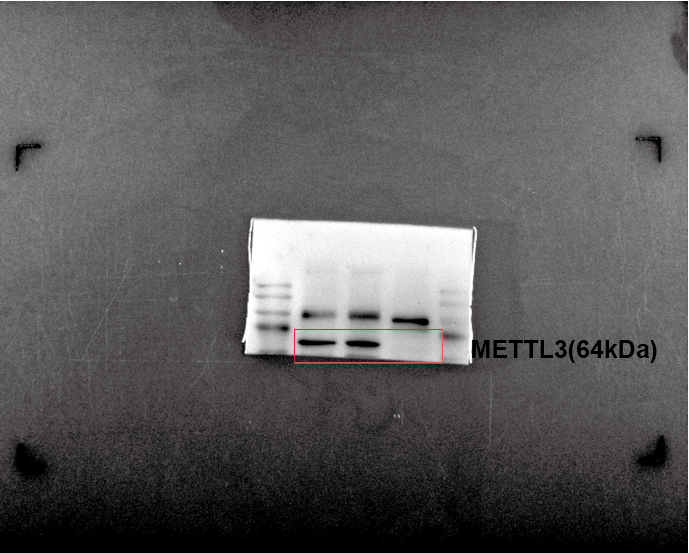

Supplement: Supplementary file 16 — The third western blot [file 41420_2022_1118_MOESM16_ESM.tif]

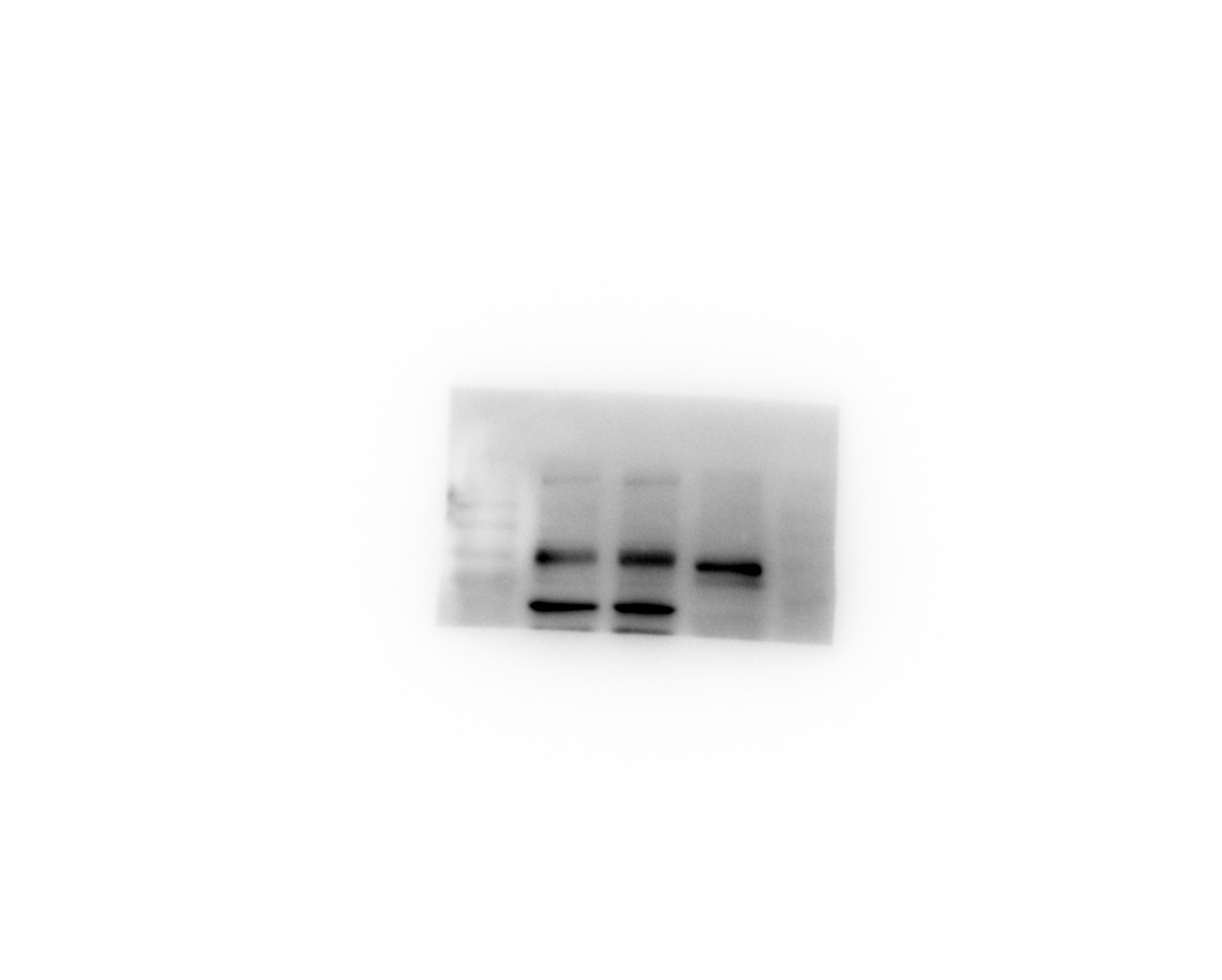

Supplement: Supplementary file 17 — The third western blot [file 41420_2022_1118_MOESM17_ESM.tif]

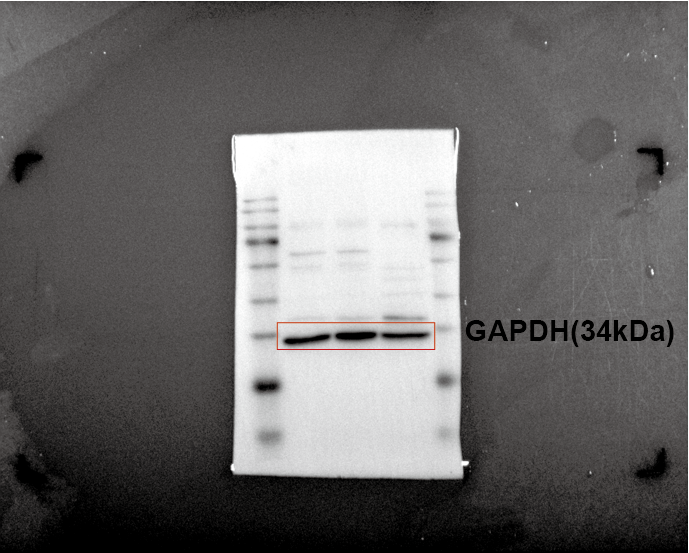

Supplement: Supplementary file 18 — The third western blot [file 41420_2022_1118_MOESM18_ESM.tif]

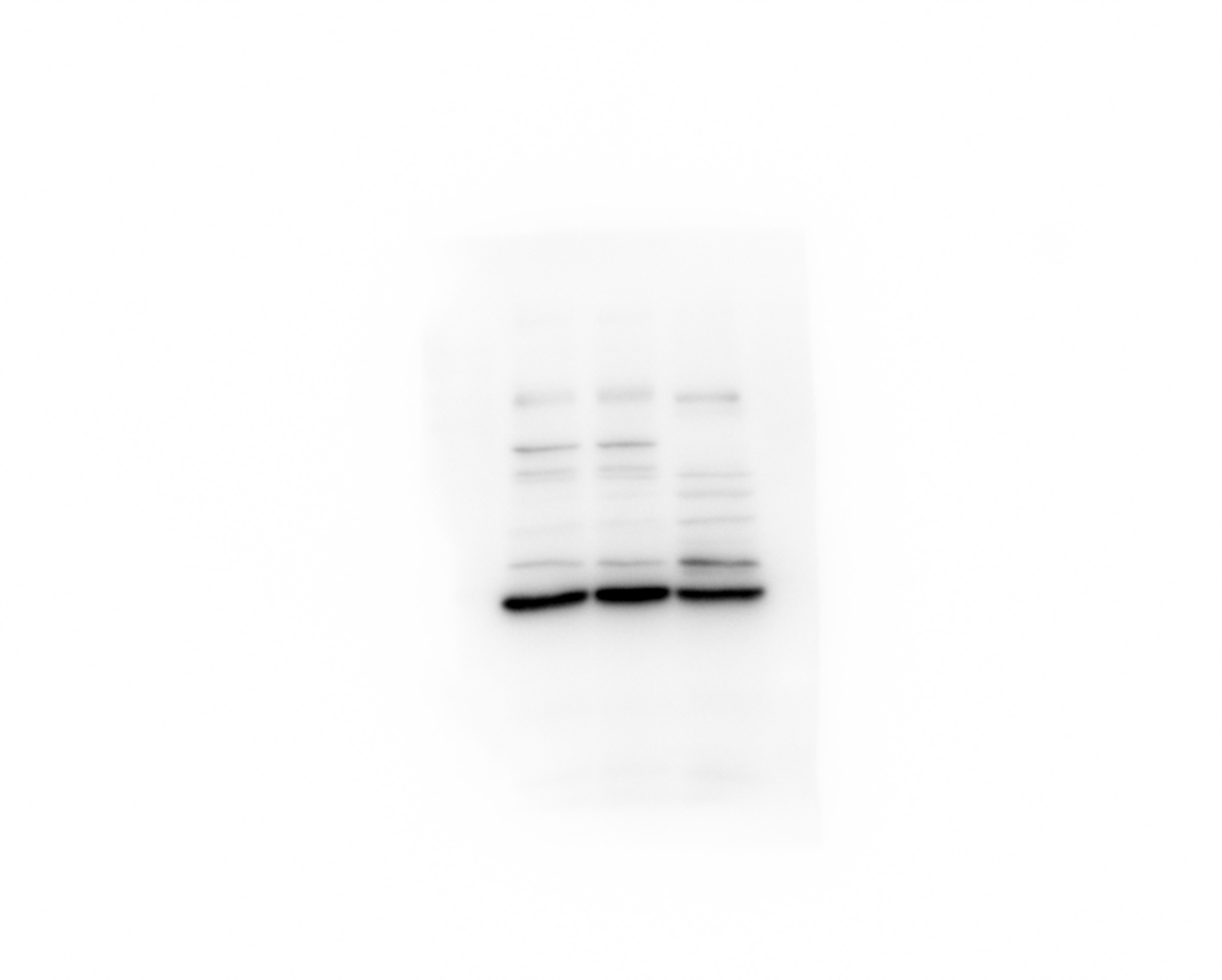

Supplement: Supplementary file 19 — The third western blot [file 41420_2022_1118_MOESM19_ESM.tif]
